# Cardiorenal Systems Modeling: Left Ventricular Hypertrophy and Differential Effects of Antihypertensive Therapies on Hypertrophy Regression

**DOI:** 10.3389/fphys.2021.679930

**Published:** 2021-06-16

**Authors:** K. Melissa Hallow, Charles H. Van Brackle, Sommer Anjum, Sergey Ermakov

**Affiliations:** ^1^School of Chemical, Materials, and Biomedical Engineering, University of Georgia, Athens, GA, United States; ^2^Clinical Pharmacology, Modeling and Simulation, Amgen Inc., South San Francisco, CA, United States

**Keywords:** cardiorenal, hypertrophy (left ventricular), angiotensin receptor antagonist, systems pharmacology, cardiac remodeling, pressure overload, volume overload, renal

## Abstract

Cardiac and renal function are inextricably connected through both hemodynamic and neurohormonal mechanisms, and the interaction between these organ systems plays an important role in adaptive and pathophysiologic remodeling of the heart, as well as in the response to renally acting therapies. Insufficient understanding of the integrative function or dysfunction of these physiological systems has led to many examples of unexpected or incompletely understood clinical trial results. Mathematical models of heart and kidney physiology have long been used to better understand the function of these organs, but an integrated model of renal function and cardiac function and cardiac remodeling has not yet been published. Here we describe an integrated cardiorenal model that couples existing cardiac and renal models, and expands them to simulate cardiac remodeling in response to pressure and volume overload, as well as hypertrophy regression in response to angiotensin receptor blockers and beta-blockers. The model is able to reproduce different patterns of hypertrophy in response to pressure and volume overload. We show that increases in myocyte diameter are adaptive in pressure overload not only because it normalizes wall shear stress, as others have shown before, but also because it limits excess volume accumulation and further elevation of cardiac stresses by maintaining cardiac output and renal sodium and water balance. The model also reproduces the clinically observed larger LV mass reduction with angiotensin receptor blockers than with beta blockers. We further provide a mechanistic explanation for this difference by showing that heart rate lowering with beta blockers limits the reduction in peak systolic wall stress (a key signal for myocyte hypertrophy) relative to ARBs.

## Introduction

Heart failure (HF) presents a major and growing health problem in developed countries. It is a leading cause of hospitalization with poor long-term survival. From 2013 to 2016, 6.2 million adults in the United States were diagnosed with HF, with estimated annual societal costs of $30.7 billion (Massie, 1998). Left ventricular (LV) hypertrophy often precedes and is a risk factor for HF and cardiovascular events (Verdecchia et al., 2001; Drazner et al., 2004). Despite significant efforts to develop better therapies, HF drug development remains a challenging task. Cardiac function is inextricably linked with renal and neurohormonal function, and insufficient understanding of effects of therapy on the integrative function or dysfunction of the heart and kidney, and subsequently on patient response and trajectory, likely contributes to both missed opportunities and wasted clinical trial resources.

The kidney is the primary controller of blood and interstitial fluid volume homeostasis, and thus plays an important role in determining both the preload and afterload experienced by the heart. The heart remodels in response to changes in loading, and the pattern of hypertrophy depends on the loading condition. Pressure overload usually causes thickening of ventricle wall, which is often observed in HF patients with preserved ejection fraction (HF-pEF). Volume overload, on the other hand, causes ventricle cavity enlargement and thinning of the ventricle wall. This pattern is typical of heart failure with reduced ejection fraction (HF-rEF). These remodeling patterns impact cardiac function, which in turn alters renal function through changes in renal perfusion, leading to changes in blood and/or interstitial fluid volume. Neurohumoral mechanisms including the renin-angiotensin-aldosterone system (RAAS), sympathetic nervous system, and natriuretic peptides regulate the integrated function of these organs.

A large number of cardiac therapies act by primarily reducing the workload on the heart, rather than by directly altering cardiac contractile function. This reduced workload is achieved by reducing heart rate, reducing peripheral resistance, and decreasing blood volume. This reduced workload translates into slowing or reversal of cardiac remodeling and improvement in overall cardiac function. Nearly all HF therapies, e.g., drugs modulating the RAAS and/or natriuretic peptides (Yusuf et al., 1991; Zannad et al., 2011; McMurray et al., 2014), beta blockers (Cadrin-Tourigny et al., 2017) and diuretics (Faris et al., 2006; Fitchett et al., 2019), achieve these effects by acting either partially or fully through the kidney by controlling water and sodium homeostasis and reducing volume retention. While these therapies have demonstrated a reduction in morbidity and mortality in HF-rEF patients, they have consistently failed to improve outcomes in trials with HF-pEF patients (Zakeri and Cowie, 2018). These failed trials indicate gaps in our understanding of how drug treatments impact cardiorenal function, even with established drugs like angiotensin receptor blockers (ARBs) and beta blockers (BBs), studies of LV hypertrophy regression have found differential effects on LV regression that are not explained solely by differences in blood pressure lowering (Devereux et al., 2004). The mechanisms underlying the differences remain incompletely understood.

Computational models in which physiology, pathophysiology, and pharmacology are described mathematically with sufficient level of mechanistic details and fidelity [an approach called quantitative systems pharmacology (QSP) modeling (Leil and Bertz, 2014; Visser et al., 2014; Peterson and Riggs, 2015)] are increasingly used to better understand and predict integrative responses to therapies (Zhang et al., 2016). QSP models can be applied to quantitatively evaluate competing hypotheses, to propose new testable hypotheses, or to understand mechanisms underlying unintuitive experimental or clinical observations. Guyton’s model, later expanded by Karaaslan et al. (2005) and Kannan and Janardhanan (2014), described in detail the human circulation system and blood pressure dynamics by modeling cardiovascular, renal, and neurohormonal regulation of hemodynamics. However, cardiac function was represented simplistically as a time averaged cardiac output, preventing simulation of such key cardiac variables as systolic/diastolic blood pressure, stroke volume, and ejection fraction. Recent updates also include the ability to simulate LV mass and remodeling, but the geometry of the heart was not considered (Summers et al., 2007). Others have developed models describing various aspects of cardiac functions which vary in scope and complexity as described in several reviews (Doshi and Burkhoff, 2016; Zhang et al., 2016). Some models utilize the concept of elastance (Suga and Sagawa, 1974), in which the relationship between ventricle volume and pressure is approximated by a single function for a wide range of heart conditions (Tsuruta et al., 1994; Stergiopulos et al., 1996; Smith et al., 2004), while the structure of the heart wall itself is not considered. This approach allows dynamic simulation of cardiac parameters, but it limits the ability to relate the dynamics of blood volume and blood pressure inside the heart ventricles to mechanical stresses in cardiac tissue, and subsequent cardiac remodeling. On the other end of the spectrum, sophisticated non-linear finite element models of beating ventricles describe the geometry of the heart and stresses within the heart wall with great detail, including the remodeling of the heart in response to loading changes (Goktepe et al., 2010; Kerckhoffs et al., 2012; Krishnamurthy et al., 2013; Genet et al., 2016). However, such models are quite expensive computationally, and they require detailed input information of heart geometry and tissue composition. Lying between these two approaches, Bovendeerd et al. (2006) derived the pressure-volume relationship for a simplified ventricle geometry as a function of stress and strain in the myocardial tissue. They also linked tissue macro characteristics to the properties of individual cardiomyocytes, thus providing a convenient step toward simulating myocardial remodeling starting at a single cell level. In parallel, the CircAdapt model (Arts et al., 2005, 2012) was developed to simulate the effects of cardiovascular adaptation and remodeling in response to changes in hemodynamics. In these models, circulation in the vascular system is usually modeled as a set of fluidic compartments, each corresponding to a group of blood vessels with distinct flow resistance, compliance, and inertia (Windkessel model) (Suga and Sagawa, 1974; Avolio, 1980; Stergiopulos et al., 1999; Tsuruta et al., 1994; Smith et al., 2004; Cole et al., 2005; Westerhof et al., 2009). However, all of these cardiac models assume a constant blood volume and do not consider the role of the kidney.

In this article, we present an integrated cardiorenal model that builds on the Guyton and Bovendeerd models to link a detailed mechanistic representation of kidney function and volume homeostasis with the dynamics of cardiac function and remodeling. We adapted the model to allow myocyte remodeling in response to changes in cardiac loading. We then applied the model to propose a mechanistic explanation for observed differences in left ventricular hypertrophy regression between two therapies, the angiotensin receptor blocker losartan and the beta blocker atenolol, that was observed in the LIFE clinical trial (Devereux et al., 2004).

## Materials and Methods

### Model Overview

**FIGURE 1:**
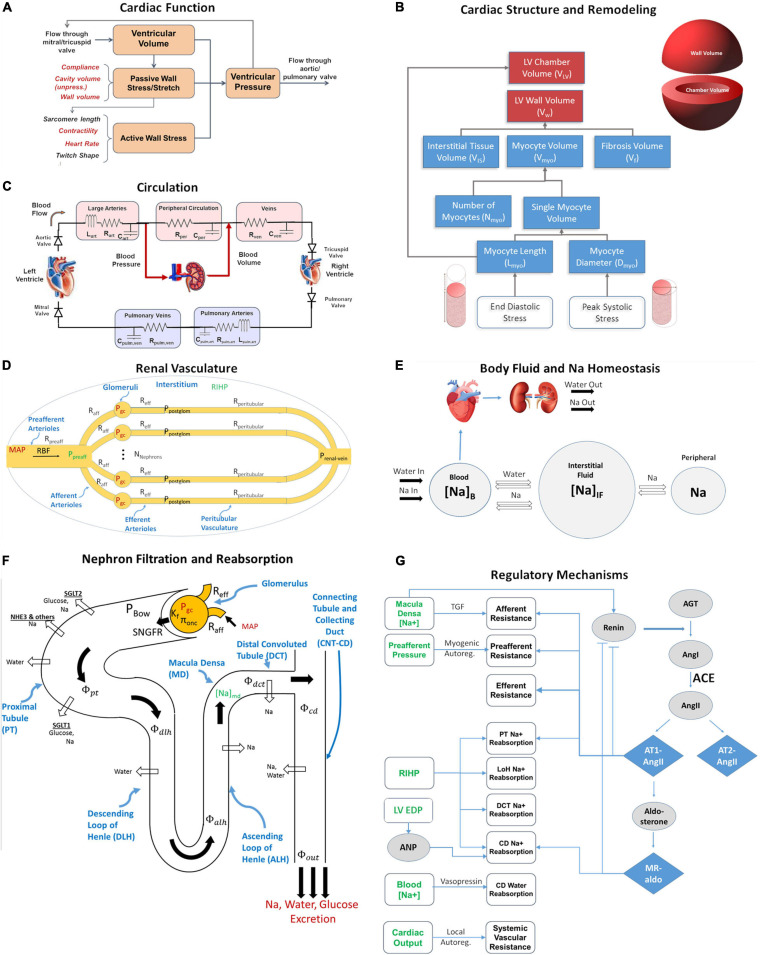
The integrated cardiorenal model links cardiac mechanics **(A)** and ventricle remodeling **(B)**, a lumped parameter description of cardiovascular circulation **(C)**, whole body Na+ and fluid homeostasis **(E)**, renal hemodynamics **(D)**, renal filtration and reabsorption **(F)**, and neurohumoral and intrinsic feedbacks including the renin-angiotensin-aldosterone system (RAAS) **(G)**. Adapted from Yu et al. (2020).

**FIGURE 2:**
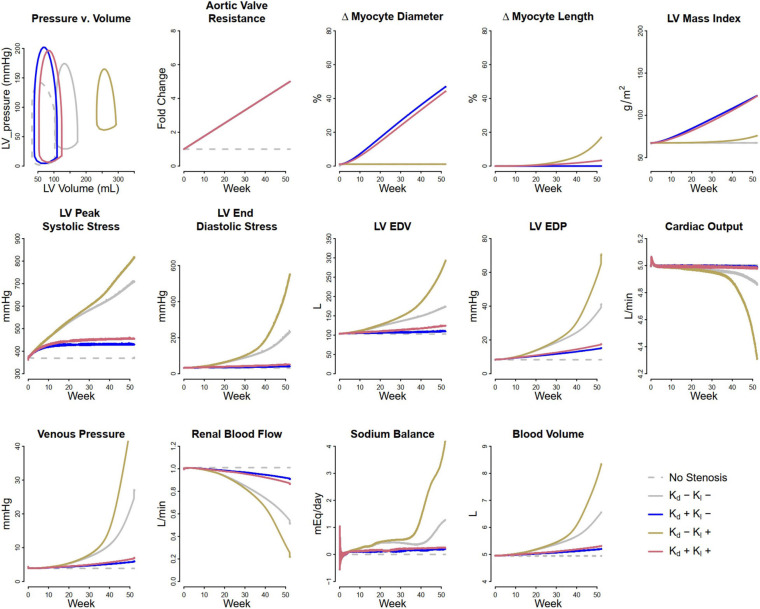
Simulated response to pressure overload due to aortic stenosis under different remodeling assumptions. Gray dashed, baseline, no stenosis; Gray solid, no remodeling; Blue, only myocyte diameter allowed to change (K_d0_ > 0, K_l0_ = 0); Brown, only myocyte length allowed to change (K_d0_ = 0, K_l0_ > 0); Red, both myocyte diameter and myocyte length allowed to change (K_d0_ and K_l0_ > 0).

The major functional modules and mechanisms of the mathematical model are shown schematically in Figure 1. We extended a previously published dynamic model of cardiac ventricular function (Arts et al., 2005; Bovendeerd et al., 2006; Figures 1A,C) to account for adaptation of myocytes and remodeling of the LV in response to changes in mechanical loading (Figure 1B). We integrated this model with our previously published model of renal function and volume homeostasis (Hallow et al., 2014, 2018; Hallow and Gebremichael, 2017b; Figures 1D–G), to allow for interaction between cardiac and renal function. The renal and cardiac portions of the model are coupled through (1) blood volume, which is regulated by kidneys through control of sodium and water excretion (Figures 1E,F), and is a key determinant of blood pressure in the circulation (Figure 1C), and (2) mean arterial pressure (time-averaged arterial blood pressure), which is calculated in the circulation submodel (Figure 1C) and is a key determinant of renal perfusion and glomerular filtration rate in the kidney model (Figures 1D,F). Model equations added or altered from previously published forms are described here. Full model equations, parameters, and initial conditions can be found in the Supplementary Material.

### Ventricular Function Model

The ventricular mechanics portion of the model was adapted from a previously published model by Arts et al. (2005) and Bovendeerd et al. (2006) (Figures 1A,C). They showed that under the assumption that myofiber structure and geometry adapt so that the myofiber shortening and tissue workload are constant, the most important structural parameter relating ventricular pressure *P*_lv_ to myocardial fiber stretch and strain is the ratio of LV wall volume V_w_ and chamber volume *V*_lv_.

(1) $$P_{l\,v}=\frac{1}{3}\,\left(\sigma_{\text{f}}-2\,\sigma_{m,r}\right)\,\ln\left(1+\frac{V_{\text{w}}}{V_{l\,v}}\right)$$

Here *σ_f_* and *σ_m,r_* are mechanical stresses in myocardium along the fiber and radial directions, respectively. This assumption allows the mathematical representation of cardiac dynamics to be substantially simplified to a set of ordinary differential equations with a limited number of parameters, without the need for finite element analysis. Right ventricle dynamics was described by the same equation with distinct *V*_w_ and *V*_rv_ parameters. In our model function of atria was not included and therefore blood was assumed to flow passively from the pulmonary vein to the left ventricle and from the vena cava to the right ventricle. Spatial variations in cardiac geometry and mechanical properties were not considered. Heart valves were modeled as ideal diodes allowing flow only in one direction, except when modeling regurgitation as described later. Detailed description of ventricular model with equations can be found in original publication (Bovendeerd et al., 2006) and in the Supplementary Material (Eqs. S66–S71).

### Cardiac Remodeling

Cardiac myocyte hypertrophy is an adaptive response of the myocardium to changing loading conditions, leading to an increase in the myocyte length and/or myocyte diameter. Grossman et al. (1975) suggested that chronically elevated peak LV systolic wall stress (pressure overload) stimulates parallel addition of contractile sarcomere units as a compensatory mechanism. This increases individual myocyte cross-sectional area without a significant change in length. On a macroscopic level the result is increased LV wall thickness (concentric remodeling) (Gaasch and Zile, 2011; Bang et al., 2014). In conditions of volume overload, when the diastolic LV wall stress is increased beyond normal values, the sarcomeres are added predominantly in series with the corresponding increase in myocytes’ length and, consequently, LV cavity diameter (eccentric remodeling) (Gaasch and Zile, 2011; Bang et al., 2014).

To describe these remodeling phenomena, the LV chamber wall volume *V*_w_ was modeled as the sum of the volume of myocytes (*V*_myo_), interstitial tissue (*V*_IS_), and fibrosis (*V*_f_).

(2) $$V_{\text{W}}=V_{\text{myo}}+V_{I\,S}+V_{\text{f}}$$

Myocytes are modeled as cylinders, and myocyte volume is determined as,

(3) $$V_{\text{myo}}=N_{\text{myo}}\,\left(\frac{\pi \,D_{\text{myo}}^{2}\,L_{\text{myo}}}{4}\right)$$

where, *N*_myo_, *D*_myo_, and *L*_myo_ are the number, average diameter, and average length of myocytes.

*D*_myo_ is the sum of the normal (healthy) diameter *D*_myo,0_ and any change in diameter Δ*D*_myo_ resulting from remodeling. *L*_myo_ is the sum of normal (healthy) length *L*_myo,0_ and any change in length Δ*L*_myo_.

(4) $$D_{\text{myo}}=D_{\mathrm{myo},0}+\Delta \,D_{\text{myo}}$$

(5) $$L_{\text{myo}}=L_{\mathrm{myo},0}+\Delta \,L_{\text{myo}}$$

As proposed by Grossman, we assume that the heart remodels to maintain a certain level of peak elastic stress *σ_f_* in the cardiac tissue fiber. The peak systolic stress *σ_f,peak,0_* under baseline conditions is used as the reference stress level. When the peak stress along the fiber *σ_f,peak_* is greater than *σ_f,peak,0_*, myocyte diameter increases, representing the formation of sarcomeres in parallel. When *σ_f,peak_* is less than *σ_f,peak,0_*, myocyte diameter decreases.

(6) $$\frac{d\,\left(\Delta \,D_{\text{myo}}\right)}{d\,t}=K_{\text{d}}\,\left(\frac{\sigma_{f,\mathrm{peak}}}{\sigma_{f,\mathrm{peak},0}}-1\right)$$

Myocytes cannot increase in diameter infinitely. As the diameter approaches the maximum value, the rate constant for the increase in diameter approaches zero:

(7) $$K_{\text{d}}=\left\{ \begin{array}{c} K_{\mathrm{d0}}^{*}\,\frac{\Delta \,D_{\text{max}}-\Delta \,D_{\text{myo}}}{\Delta \,D_{\text{max}}},\sigma_{f,\mathrm{peak}}\geq \sigma_{\mathrm{peak},0}\\ K_{\mathrm{d0}}\;\sigma_{f,\mathrm{peak}}<\sigma_{f,\mathrm{peak},0} \end{array}\right.$$

Δ*D*_max_ is assumed to be equal to *D_myo,0_*, so that the increase in diameter is limited to 2X normal, based on the upper limit of histologic observations of myocyte diameter (Olivetti et al., 1994, 1996; Tracy and Sander, 2011). *K*_d0_ was determined by fitting clinical data on LV hypertrophy regression, as described later.

Similarly, we assume that myocytes add sarcomeres in series and thus increase in length when LV passive stress along the fiber at the end of diastole is elevated. LV end diastolic (ED) stress under baseline conditions is used as the reference stress level *σ_f,ED,0_*. When the ED passive stress along the fiber *σ_f,ED_* is greater than *σ_f,ED,0_*, myocyte length increases, representing the formation of sarcomeres in series.

(8) $$\frac{d\,\left(\Delta \,L\right)}{d\,t}=K_{l}\,\left(\frac{\sigma_{f,\mathrm{ED}}}{\sigma_{f,\mathrm{ED0}}}-1\right)$$

(9) Kl={Kl⁢0*⁢Δ⁢Lmax-Δ⁢LΔ⁢Lmax,σf,ED≥σf,ED,0             0   σf,ED<σf,ED, 0

Δ*L*_max_ is assumed to be equal to *L*_myo,0_, so that the increase in length is limited to 2X normal. *K*_l0_ was determined by fitting clinical data on LV hypertrophy regression, as described later.

The left ventricle cavity volume at zero transmural pressure increases as the myocyte length increases, according to the equation,

(10) $$V_{L\,V,c\,a\,v\,i\,t\,y}=V_{L\,V\,0}\,{\left(1+\frac{\Delta \,L}{L_{\mathrm{myo},0}}\right)}^{3}$$

where *V*_LV0_ is the cavity volume before any remodeling has occurred.

### Circulation Model

The systemic and pulmonary circulation are described with a lumped parameter model, as illustrated in Figure 1C. The systemic circulation includes the left ventricle (LV) and three vascular beds: the arterial bed consisting of large and medium size arteries; the peripheral bed containing smaller arteries, arterioles, and capillaries; and the venous bed, with venules and medium and large veins. Since the primary focus of this paper is the systemic circulation, the pulmonary circulation is implemented more simplistically. The arterial and peripheral beds are lumped together into one arterial bed. Thus, the pulmonary circulation is comprised of the right ventricle (RV), and arterial and venous beds. Each bed is modeled as a compartment, with volume *V* as a state variable, and by parameters, hydraulic resistance *R*, vessel capacitance *C* and, in case of systemic and pulmonary arterial beds, inertance *I*.

For each compartment *i*, the change in its volume *V*_I_ is determined by the balance between the blood inflow, *Q*_I–1_, and blood outflow *Q*_I_.

(11) $$\frac{d\,V_{i}}{d\,t}=Q_{i-1}-Q_{i}$$

Blood flow *Q*_I_ between compartments is calculated from eq. 12, in which the inertance *I*_I_ equals zero for all *i*, except for the systemic and pulmonary arterial beds (Figure 1C).

(12) $$\frac{I_{i}}{R_{i}}\cdot \frac{d\,Q_{i}}{d\,t}+Q_{i}=\frac{P_{i-1}-P_{i}}{R_{i}}$$

Heart valves control the flow in and out of the LV and RV. They are assumed to be non-leaky, with blood flowing forward when the pressure in the upstream compartment P_I–1_ is greater than in the downstream compartment P_I_, (P_I–1_ > P_I_); and no flow otherwise (P_I–1_ < P_I_). All blood compartments except LV and RV are considered passive and pressure inside them is calculated as,

(13) $$P_{i}=\frac{V_{i}-V_{i}^{0}}{C_{i}}$$

where V_i_^0^ is the volume when the system is at equilibrium with environment without cardiac activity. Total blood volume *V*_b_ in the system is equal to the sum of all compartment volumes.

(14) $$V_{b}=\sum_{i}V_{i}$$

### Renal Function Model

The renal function portion of the model is summarized in Figures 1D–G, and has been described in detail previously (Hallow and Gebremichael, 2017a,b). Full model equations are provided in the Supplementary Material. Briefly, this model describes key physiological processes involved in renal function and in maintaining Na^+^ and water homeostasis. It describes blood flow, resistance, and pressures through the renal vasculature (Eqs. S1–S4); Starling forces across the glomerulus (Eq. S5); filtration of water, glucose, and Na^+^ through the glomerulus (Eqs. S5–S7, S11); reabsorption of glucose through sodium glucose co-transporters (SGLT) along the proximal tubule (Eqs. S8, S9) and urinary glucose excretion (Eq. S10); reabsorption of Na+ through SGLT, NHE3, and other mechanisms in the proximal tubule (Eqs. S12–S15) and along the rest of the nephron and urinary Na+ excretion (Eqs. S16–S18); reabsorption of water across osmotic gradients along the nephron (Eqs. S19–S27) and urinary water excretion (Eq. S28); tubular hydrostatic pressures along the nephron (Eqs. S37–S40); whole body balance of Na+ and water, and distribution between blood, interstitial, and peripheral compartments (Eqs. S29–S36).

The model incorporates multiple intrinsic and neurohormonal regulatory feedback mechanisms. (1) Vasopressin is modeled as a function of plasma Na^+^ concentration (Eq. S45) that alters collecting duct water reabsorption (Eq. S25). (2) The pressure-natriuresis phenomenon is modeled as a signal from renal interstitial hydrostatic pressure (Eqs. S46, S47) that alters Na^+^ reabsorption rates along the nephron (Eqs. S14, S16). (3) Tubuloglomerular feedback (TGF) is modeled as a signal from macula densa sodium flow (Eq. S48) that signals the afferent arteriole (Eq. S1) to constrict or relax. (4) Myogenic autoregulation is modeled as a function of preafferent pressure (Eqs. S49, S50) that signals the preafferent arterioles (Eq. S1) to constrict or relax. (5) Whole-body blood flow autoregulation is modeled as a signal from cardiac output that modulates peripheral resistance (Eqs. S12, S51) (6) Atrial Natriuretic Peptide (Eqs. S61, S62). (7) To describe the renin-angiotensin-aldosterone System (RAAS), renin secretion is modeled as a function of macula densa sodium flow, with a strong inhibitory feedback from angiotensin II bound to the AT1 receptor (AT1-bound AngII) (Eqs. S52–S55). Renin generates Angiotensin I, which can be converted to Angiotensin II by ACE or chymase, or degraded (Eq. S56). Angiotensin II can bind to the AT1 or AT2 receptor, or can be degraded (Eq. S57). AT1-bound AngII signals efferent, preafferent, and afferent vasoconstriction, proximal tubule sodium retention, and aldosterone secretion (Eqs. S58, S59). Aldosterone binds to the mineralocorticoid receptor (Eq. S60) and signals sodium retention in the connecting tubule/collecting duct (Eq. S61).

### Linking the Cardiac and Renal Models

The cardiac and renal models are linked together through mean arterial pressure (MAP) and blood volume. MAP is calculated from the arterial pressure wave generated by the cardiac model, and used as an input into the kidney model, to calculate renal blood flow and glomerular hydrostatic pressure (Eqs. S3, S4). The renal model determines sodium and water excretion rates. The excess of water excretion over water intake decreases the fluid returned through the renal vein into the venous bed, that in turn determines total blood volume V_b_ (Eq. S29). However, venous bed volume is already defined in the section “Circulation Model” (Eq. 11). In order to reconcile two values of venous volume, its value resulting from Renal Model is now called “target” venous volume *V*_ven,target_. It accounts for the mismatch between the total blood volume resulting from the renal model and volume of all the other compartments from the circulation model.

(15) $$V_{v\,e\,n,t\,a\,r\,g\,e\,t}=V_{b}-V_{L\,V}-V_{\text{art}}-V_{\text{per}}-V_{R\,V}-V_{\mathrm{pulm},\mathrm{art}}-V_{\mathrm{pulm},\mathrm{ven}}$$

Equation 11 for venous volume is then altered allowing the volume to adjust and reach this target volume:

(16) $$\frac{d\,V_{v\,e\,n}}{d\,t}=Q_{\mathrm{per}-\mathrm{ven}}-Q_{t\,r\,i\,c\,u\,s\,p\,i\,d}+{\text{k}}_{\text{ven},\text{target}}\,\left(V_{v\,e\,n}-V_{v\,e\,n,t\,a\,r\,g\,e\,t}\right)\,$$

### Software Implementation

The model was implemented as a system of ordinary differential equations and all simulations were conducted using the RxODE package (Wang et al., 2016) in R version 3.6.0. The model can be accessed at https://bitbucket.org/hallowkm/lv-hypertrophy-model.

### Modeling Approach

#### Establishing Baseline Model Behavior

The normal steady-state outputs of the new combined model were compared with normal ranges previously obtained with the separate cardiac and renal models. We further tested the model to ensure that under normal baseline conditions, the model was in a stable steady state, that all regulatory signals were at their set point, that all hypertrophic signals were zero, and that the model responded appropriately to perturbations including changes in cardiac output (CO) setpoint and altered Na+ intake (Hallow and Gebremichael, 2017b). Lastly, we ensured that the blood pressure responses to therapies previously implemented in the renal model were still reproduced appropriately. These validation tests are summarized in the Supplementary Figure 1.

#### Modeling Response to Pressure and Volume Overload

Grossman et al. (1975) previously compared experimentally measured hemodynamic and structural parameters [LV mass index, LV wall thickness, LV internal diameter, LV end diastolic pressure (EDP), and LV peak systolic pressure (PSP)] between normal subjects, subjects with pressure overload primarily due to aortic valve disease, and subjects with volume overload primarily due to mitral regurgitation. To reproduce this study, aortic valve disease and pressure overload was simulated by increasing R_art_, which encompasses the resistance of the valves and larger arteries. Mitral valve regurgitation was simulated by allowing back-leak across the mitral valve when the pressure difference across the valve exceeds a certain value ΔP_leak_.

(17) $$Q_{\text{mitral}}=\left\{ \begin{array}{c} \frac{P_{\mathrm{plum},\mathrm{ven}}-P_{L\,V}}{R_{\text{mitral}}}\;P_{\mathrm{plum},\mathrm{ven}}>P_{L\,V}\\ \frac{P_{\mathrm{plum},\mathrm{ven}}-P_{L\,V}}{R_{\text{mitral}}}\;\left(P_{L\,V}-P_{\mathrm{plum},\mathrm{ven}}\right)>\Delta \,P_{l\,e\,a\,k}\\ 0\;\text{otherwise} \end{array}\right.$$

The stress thresholds *σ_f,peak,0,_ σ_f,ED,0_* and relative values of the rate constants governing changes in myocyte diameter *K*_d0_ and myocyte length *K*_l0_ were determined by fitting the measured values in the study.

#### Simulating the LIFE Study

The LIFE study reported changes in LV hypertrophy over time in patients with essential hypertension treated with either losartan or atenolol (Devereux et al., 2004). To simulate this study, we produced an LV hypertrophy virtual patient by inducing hypertension as previously described in ref. (Hallow et al., 2014; Hallow and Gebremichael, 2017a), and simulated the response to losartan and atenolol.

We have previously described the calibration and validation of the plasma renin concentration (PRC) as RAAS biomarker and blood pressure response to treatments targeting the RAAS, including losartan (Hallow et al., 2014). We confirmed that the previous model calibration still held in the updated model (Supplementary Figure 2). Briefly, 100 mg losartan is modeled as a sustained 92% blocking of the AT1 receptor. As illustrated in Figure 1, this leads to reduced renal vascular resistances, reduced PT Na+ reabsorption, reduced aldosterone secretion, and increased renin secretion. Diurnal variations in inhibition were not considered.

The beta blocker atenolol was modeled as a reduction in heart rate of 8 beats per minute, as observed in the LIFE study, as wells as a reduction in renin secretion sufficient to reduce renin activity by 40% (Wilkinson et al., 1980; Dreslinski et al., 1982). Because of the nonlinear feedback on renin secretion when the RAAS is blocked, a 90% inhibition of renin secretion was required to produce a 40% reduction in renin activity. This degree of RAAS inhibition, coupled with the heart rate effect, was sufficient to produce the blood pressure reduction observed in LIFE study, and thus no further effect of atenolol on renal vascular resistance was included.

## Results

### Baseline Model Behavior

Model parameters added or changed from previously published model versions (Bovendeerd et al., 2006; Hallow et al., 2014, 2018) are given in Table 1. Full parameters and initial conditions are given in Supplementary Tables 1–5. Using these parameters as input, the model is run until the steady state solution is achieved, which is later considered as a baseline. As shown in Table 2, the baseline behavior of the model is consistent with cardiac, vascular, and renal function in healthy humans.

**TABLE 1:** Cardiac model parameters.

**Parameter**	**Definition**	**Value**	**Units**	**Source**
Δ*d*_max_	Maximum increase in myocyte diameter	25	μm	Olivetti et al., 1994, 1996; Tracy and Sander, 2011
Δ*l*_max_	Maximum increase in myocyte length	115	μm	Olivetti et al., 1994, 1996; Tracy and Sander, 2011
σ_f, ED,0_	End diastolic stress threshold for eccentric remodeling	4.5	kPa	End diastolic stress under baseline conditions
σ_f,peak,0_	Peak systolic stress threshold for concentric remodeling	49.2	kPa	Peak systolic stress under baseline conditions
D_myo0_	Myocyte diameter	23.3	μm	Calculated from N_myo_, L_myo0_, V_wo_ (Olivetti et al., 1994, 1996; Zafeiridis et al., 1998; Tracy and Sander, 2011)
HR	Heart rate	70	Beats/min	
k_ven,target_	Rate constant for venous volume link renal and cardiac submodels	1	/min	
K_d0_	Rate constant for the increase in myocyte diameter in response to peak systolic stress	43.8	μm/year	Estimated
K_l0_	Rate constant for the increase in myocyte length in response to end diastolic stress	17.5	μm/year	Estimated
L_myo0_	Myocyte length	115	μm	Olivetti et al., 1994, 1996; Zafeiridis et al., 1998; Tracy and Sander, 2011
N_myo_	Number of myocytes	3.3e9		Olivetti et al., 1994, 1996; Tracy and Sander, 2011
V_f_	LV fibrosis volume	4.8	mL	Beltrami et al., 1994; Olivetti et al., 1994
V_IS_	LV interstitial tissue volume	26.4	mL	Beltrami et al., 1994; Olivetti et al., 1994
V_LV0_	Unpressurized LV chamber volume	52	mL	Beltrami et al., 1994; Olivetti et al., 1994
V_w0_	LV wall volume	120	mL	Beltrami et al., 1994; Olivetti et al., 1994

**TABLE 2:** Key model variables at baseline fall within the physiologically normal range.

**Biomarker**	**Units**	**Normal**	**Model**
		**range**	**output**
Systolic blood pressure	mmHg	100–140	113
Diastolic blood pressure	mmHg	60–90	71
Ejection fraction	%	55–75	69
Cardiac output (resting)	L/min	4–8	5.0
LV mass	Grams	100–180	126
LV end diastolic pressure (LV EDP)	mmHg	5–12	8.2
LV peak systolic pressure (LV PSP)	mmHg	100–150	140
LV end diastolic volume (LV EDV)	mL	75–150	103
LV end systolic volume (LV ESV)	mL	25–60	32
Stroke volume (SV)	mL	60–100	71
Mean pulmonary arterial pressure	mmHg	9–20	12.7
Heart rate (resting)	Bpm	50–82	70
Glomerular filtration rate (GFR)	mL/min	80–130	100
Renal blood flow (RBF)	mL/min	600–1200	1,000
Interstitial fluid volume	L	9–15	12
Blood volume	L	4–6	4.95
Total peripheral resistance	mmHg-min/L	13–19	16.9

### Hypertrophic Response to Pressure Overload

Pressure overload was simulated as a consequence of aortic stenosis, which was modeled by increasing systemic arterial resistance R_art_ by five-fold over the course of the simulation. The threshold peak systolic stress, σ_f,peak,0_ above which myocytes increase in diameter was set as the baseline peak systolic stress of 49.2 kPa (369 mmHg), while the threshold end diastolic stress σ_f,ED,0_ above which myocytes increase in length was set at 4.5 kPa (33.75 mmHg). The latter is slightly above the baseline level of 4.3 kPa (32.25 mmHg), since small changes in volume status do not cause irreversible changes in myocyte length. Figure 2 shows the simulated evolution of key model variables over the course of 1 year, in response to aortic stenosis. Lines representing baseline behavior without aortic stenosis are co-plotted with four hypothetical remodeling scenarios: (1) no change in myocyte length and diameter, (2) changes in myocyte length only, (3) changes in myocyte diameter only, or (4) concurrent changes in diameter and length. When no remodeling was allowed (Case 1: K_d_ = 0 and K_l_ = 0 – solid gray curves), LV peak systolic stress (LV PSS) greatly increased over time. Blood volume began to accumulate as sodium and water were retained, reflected by positive water and sodium balance, in order to maintain cardiac output. This resulted in greatly elevation of ED stress and EDP to physiologically unsustainable levels that would likely result in systemic and pulmonary edema and the signs and symptoms of heart failure.

If instead, the myocytes were allowed to increase in diameter (Case 2: non-zero K_d_, K_l_ = 0 – blue curves), LV PSS rose initially but then returned to near normal values. There was a very small rise in blood volume and no change in LV ED stress, as sodium and water balance were maintained. Thus, myocyte thickening appears to be a beneficial adaptive response that not only normalizes cardiac wall stress but also sustains cardiac output and allows the kidney to maintain sodium and water balance.

On the other hand, if myocytes were not allowed to increase in diameter, but were allowed to elongate in response to elevated ED stress (Case 3: K_d_ = 0, non-zero K_l_, – brown curves), the response was maladaptive: there was additional fluid retention even beyond case 1, as well as greatly elevated LV EDP and ED stress, and further rightward shifting of the pressure-volume (P-V) loop. In this physiologically untenable state, the limits of the model system’s ability to maintain cardiac output were reached.

When both remodeling mechanisms were allowed (Case 4: non-zero K_d_ and K_l_ – red curves), the response fell between case 1 and case 2. LV peak stress returned near normal, but with some sustained fluid retention, LV ED stress elevation, and rightward shifting of the P-V loop.

**FIGURE 3:**
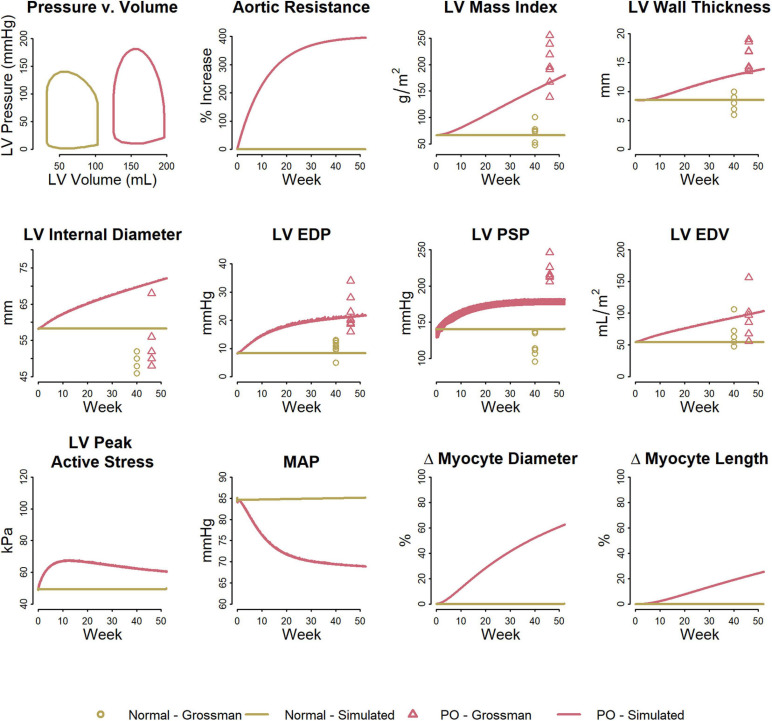
Model reproduces observed patterns of hypertrophy in pressure overload due to aortic stenosis. Data from Grossman et al. (1975).

Figure 3 shows the fitted response to pressure overload due to aortic stenosis observed by Grossman et al. (1975). The magnitude of increase in R_art_ (three-fold) was determined to best fit the absolute increase in LV mass index and LV peak systolic pressure. The ratio of the remodeling rate constants K_d_ to K_l_ (Table 1) was determined to best fit the change in wall thickness and ED stress. The model is able to reproduce salient differences between control and pressure-overloaded subjects in LV mass index (LVMI), LV wall thickness, LV EDP, LV PSP, and LV end diastolic volume (EDV). Because the model approximates the LV as a sphere, while its true shape is more ellipsoidal, the simulated internal diastolic diameter is larger than the measured value at both baseline and after remodeling, but the magnitude of increase agrees with the data. The simulated myocyte diameter increase was 75% while myocyte length increase was 25%. This combination of concentric and eccentric remodeling during pressure overload allowed PSS to return toward normal, but LV EDP and LV PSP remained elevated.

### Hypertrophic Response to Volume Overload

**FIGURE 4:**
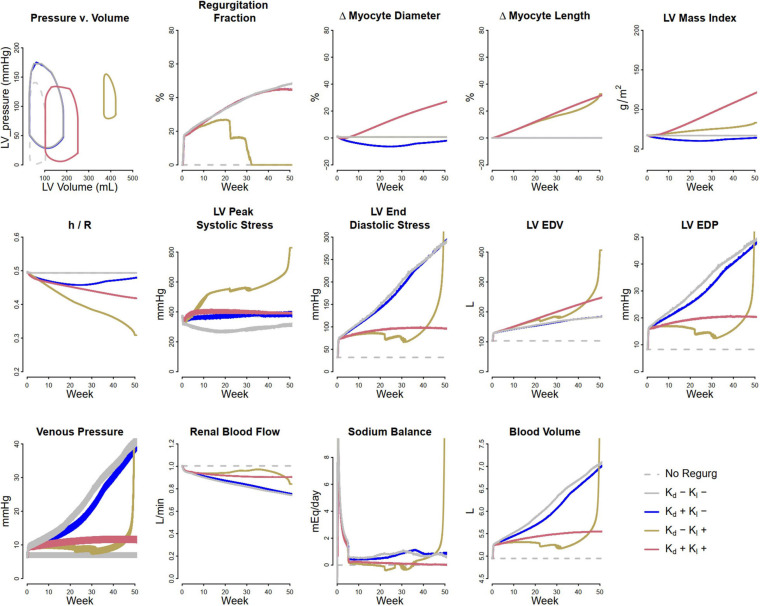
Simulated response to volume overload due to mitral regurgitation under different remodeling assumptions. Gray dashed, baseline; Gray solid, no remodeling; Blue, only myocyte diameter allowed to change (K_d0_ > 0, K_l0_ = 0); Brown, only myocyte length allowed to change (K_d0_ = 0, K_l0_ > 0); Red, both myocyte diameter and myocyte length allowed to change (K_d0_ and K_l0_ > 0).

Volume overload was simulated as a consequence of progressive development of mitral regurgitation. To model mitral regurgitation, ΔP_leak_ (leak pressure for the mitral valve) was decreased over the course of the simulation from 17.5 kPa (131.25 mmHg) to 12.5 kPa (93.75 mmHg). Figure 4 shows the simulated response. The first panels show the Pressure-Volume (P-V) loop at the end of 1 year of mitral regurgitation. The remaining panels show evolution of several model parameters over the course of 1 year. When no remodeling was allowed (Case 1: K_d_ = 0 and K_l_ = 0 – solid gray curves), LV EDP and blood volume both greatly increased over time, resulting in an unsustainable state that would likely result in systemic and pulmonary edema and progressive signs of heart failure. This fluid retention occurred because elevated venous pressure reduced renal perfusion and produced a sodium imbalance that caused volume accumulation. Allowing myocyte diameter to change in response to LV PSS (Case 2: non-zero K_d_, K_l_ = 0 – blue curves) did little to mitigate this. Since LV PSS was initially reduced (as the back leak during systole reduced the peak pressure generated by the ventricle), myocytes decreased in diameter and the ratio of wall thickness to chamber radius (h/R) fell, and LV EDP was slightly reduced, but LV ED stress was actually increased. If myocytes were not allowed to increase in diameter, but were allowed to increase in length in response to elevated ED stress (Case 3: K_d_ = 0, non-zero K_l_ – brown curves), the increasing chamber volume accommodated the excess preload for a while, and increases in LV EDP, venous pressure, and blood volume were initially limited. However, peak systolic stress was elevated. At the same time, as the heart wall thinned (h/R ratio fell), the pressure generated by the heart during systole fell, and by 30 weeks did not even exceed the pressure necessary to produce regurgitation (2nd panel). As it thinned further, the system entered an unstable state in which cardiac output fell while venous pressure rose dramatically, renal perfusion fell and the sodium imbalance became large, and blood volume rose rapidly. The PV loop shifted dramatically up and to the right, as stroke volume and ejection fraction plummeted. However, when both remodeling mechanisms were allowed (case 4, K_d_ ≠ 0, K_l_ ≠ 0 – red curves), increases in LV EDP and blood volume were limited compared to case 1 and 2, and the rightward shift of the PV loop was limited and stroke volume and ejection fraction were preserved relative to case 3. This suggests that the combined effects of eccentric and concentric remodeling may help to stabilize cardiac wall stress, maintain stroke volume, and mitigate but not prevent volume accumulation.

**FIGURE 5:**
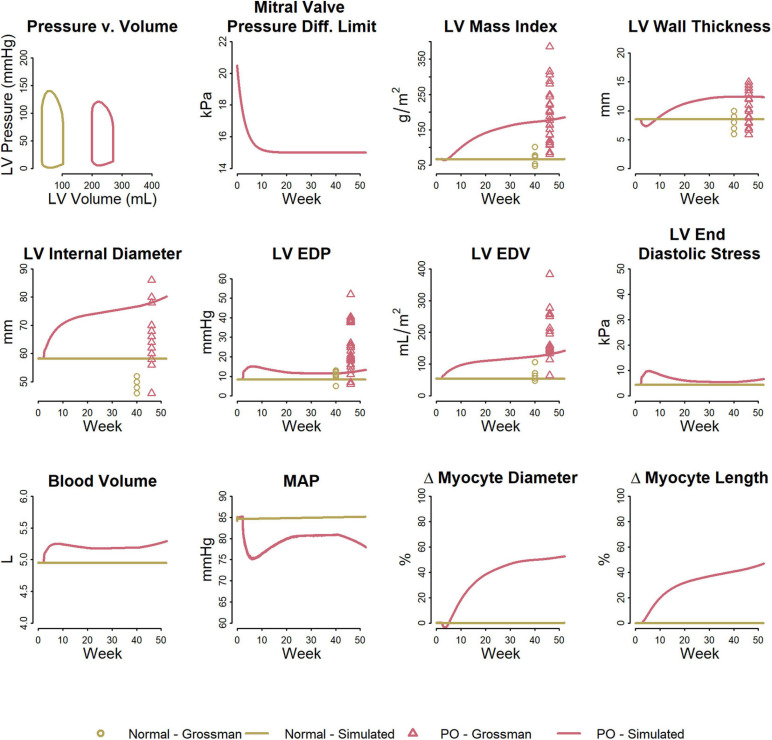
Model reproduces observed patterns of hypertrophy in volume overload due to mitral regurgitation. Data from Grossman et al. (1975).

Figure 5 shows the fitted response to volume overload due to mitral regurgitation observed by Grossman et al. (1975). The magnitude of decrease in ΔP_leak_ (top row, second panel) was determined to best fit the absolute increase in LV mass index. The ratio of the remodeling rate constants K_d_ to K_l_ (Table 1) were fixed to the value determined when simulating pressure overload, as described above. The model was able to reproduce salient differences between control and volume-overloaded subjects in LVMI, LV wall thickness, LV EDP, and LV EDV. As with the pressure-overload case, the simulated internal diastolic diameter was larger than the measured value at both baseline and after remodeling, but the magnitude of increase was in agreement with the observed data. Simulated myocyte diameter increase was 75% and myocyte length increase was 70%. Blood volume increased moderately. The increase in myocyte length caused a rightward shift in the PV loop as the LV chamber dilated. Since LV ED stress returned to near baseline, but was not completely normalized, this progression would likely continue.

### Effect of Antihypertensive Therapy on Regression of LV Hypertrophy

The analysis of pressure and volume overload described above allowed determination of the ratio of time constants for concentric/eccentric remodeling (Kd/Kl), but did not permit determination of the absolute time constants, since LV geometry was not measured over time in those studies.

**FIGURE 6:**
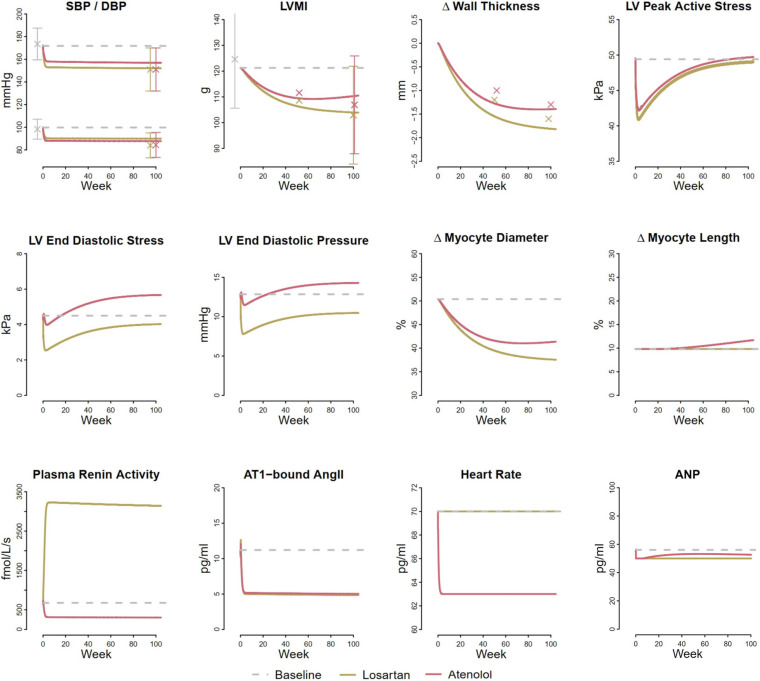
Model reproduces larger reduction in LVMI and wall thickness with losartan compared to atenolol observed in the LIFE clinical trial (Devereux et al., 2004). Both treatments reduce LV peak active stress, but losartan reduces LV end diastolic stress, while atenolol increases it.

To determine K_d_, we fit the LV hypertrophy regression observed with antihypertensive treatment in the LIFE study, under the assumption that both increases and decreases in myocyte diameter in response to changes in load can be described by the same time constant. Figure 6 shows results of the simulated LIFE study as a response to the therapy by a representative virtual patient, along with measured clinical data when available. The model slightly underpredicted the reduction in SBP/DBP with each treatment. Since LIFE did not include a true placebo group, the reported blood pressure drop due to treatment may be overestimated. The modeled blood pressure response to losartan was previously calibrated using placebo-adjusted data (Hallow et al., 2014), and this calibration was not altered for this simulation. Atenolol was modeled as a reduction in heart rate of 7 beats/min, as observed in LIFE, and a decrease in renin secretion sufficient to decrease plasma renin activity (PRA) by 50% (Dietz et al., 2008). These two effects were sufficient to produce blood pressure reductions matching those of losartan (top row, left panel), without further adjustments.

The model predicted similar blood pressure reductions but larger reductions in LV mass with losartan than atenolol, in agreement with findings from the LIFE trial, as well as several meta-analyses of LV hypertrophy regression (Klingbeil et al., 2003; Devereux et al., 2004; Fagard et al., 2009). The smaller reduction in LV mass with atenolol occurred because atenolol produced a smaller reduction in both LV peak active stress and end diastolic stress than losartan, even though the mean arterial pressure reduction was the same. While the therapies have different effects of plasma renin activity, they produce similar reductions in AT1-bound Ang II and atrial natriuretic peptide (ANP).

**FIGURE 7:**
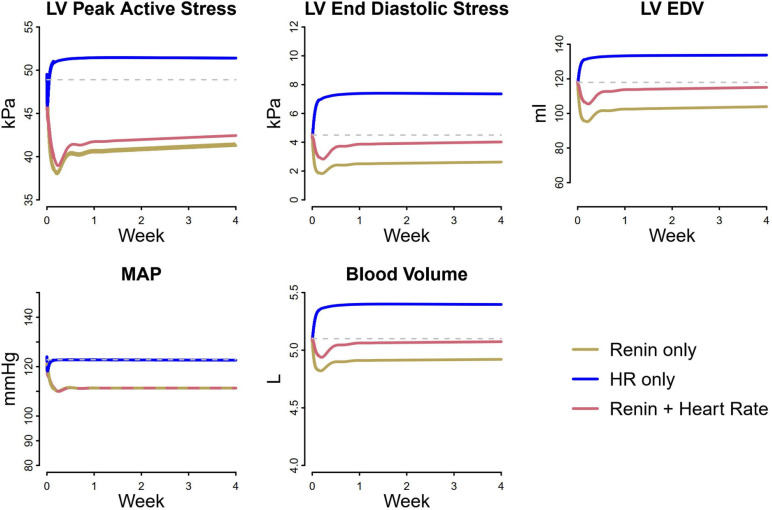
Differential analysis of pathways affected by losartan and atenolol on cardiovascular biomarkers. Renin suppression lowers LV peak active stress, end diastolic stress, and end diastolic volume, due to decreased MAP and blood volume. In contrast, heart rate initially decreases LV peak active stress and MAP, but causes an increase in blood volume, returning MAP to its original value and increasing LV peak active stress, end diastolic stress, and end diastolic volume. When heart rate lowering is combined with renin suppression, the resulting reduction in LV stresses is less than with renin suppression alone, even though the blood pressure reduction is the same.

We next sought to understand the reason for this smaller reduction in stresses with atenolol. Losartan acts by blocking the AT1 receptor, preventing angiotensin II from binding. This results in renal vasodilation (especially of the efferent arteriole), reduced tubular sodium reabsorption, and subsequently natriuresis and diuresis that serves to lower blood pressure. Atenolol inhibits the RAAS by blocking renin secretion, but also affects the heart directly by reducing heart rate. As shown in Figure 7, inhibiting renin secretion alone causes a natriuretic response that lowers MAP, reducing both LV peak active and end diastolic stress. On the other hand, while reducing heart rate initially reduces MAP and LV peak active stress, it also reduces sodium excretion, leading to fluid retention that restores MAP, increases filling volume (LV EDV), and raises both LV peak systolic and end diastolic stress. This suggests that the stress-reducing effects of renin inhibition and subsequent natriuresis/diuresis with beta blockers are opposed by the stress-increasing effects of heart rate changes. Consequently, the net change in stress and thus the observed reduction in LV mass and wall thickness are lower than with therapies, like losartan, that act primarily by natriuresis/diuresis.

## Discussion

Cardiac and renal function are hemodynamically and neurohormonally linked. The integrated model of cardiac and renal function described here provides a tool for investigating and understanding the impact of dysfunction or intervention in one organ on the function of the other in this critical organ system.

The model reproduces different LV hypertrophic patterns observed due to mitral regurgitation and aortic stenosis. The cardiac remodeling rules utilized are similar to those proposed by Grossman et al. (1975) and implemented by Arts et al. (1994). The novelty of the current model is that, by integrating cardiac and renal function, the model naturally captures the effects of changes in cardiac function on renal perfusion, sodium and water retention, and subsequently on blood volume, preload, and afterload of the heart. The simulations indicate that concentric remodeling in response to aortic stenosis is adaptive (from hemodynamics standpoint) not only because it normalizes ventricle wall stress [as others have shown before (Lee et al., 2016)], but also because it helps to restore renal perfusion, minimizing blood volume accumulation and preventing elevation in preload. On the other hand, eccentric remodeling is maladaptive and contributes to volume retention and elevation in preload. Fitting to clinical measurements by Grossman et al. (1975), our simulations confirm that the response to pressure overload due to aortic stenosis is dominated by concentric remodeling with some contribution from eccentric remodeling.

In response to mitral regurgitation, the simulations indicate that eccentric remodeling may initially help mitigate volume overload, but because this type of remodeling is not self-limiting (i.e., chamber dilatation does not help normalize LV end diastolic stress), as the ventricle expands and is unable to maintain a normal output, fluid begins to accumulate at an accelerated rate (relative to the case when no remodeling occurs, see Figure 4). Concentric remodeling alone is not able to prevent this volume accumulation, but when coupled with eccentric remodeling, it helps to stabilize blood volume and slow the rise in preload. Fitted to the clinical data of Grossman et al. (1975), our simulations confirm that the response to volume overload with mitral regurgitation includes both eccentric (increase in myocyte length) and concentric (increase in myocyte diameter) remodeling.

By connecting system level models of cardiac and renal function, complemented with effects of the renin angiotensin aldosterone system, our integrated model provides a tool for evaluating mechanistic hypotheses and understanding effects of various therapies on cardiac and renal health. Our simulations were able to demonstrate the differential effect of losartan and atenolol on LV hypertrophy regression, providing a plausible mechanistic explanation for this difference. We showed that while the natriuretic and antihypertensive effects of atenolol are comparable to losartan, the heart rate effect of atenolol counters its antihypertensive effect on lowering LV peak systolic and end diastolic stress, thus producing a smaller net reduction in LV mass. Of course, there are likely other cardiac benefits of heart rate lowering with beta blockers that are not considered in this model (Kubon et al., 2011).

In this work, we attempted to provide a simple mechanistic model of cardiac remodeling that is able to reproduce some clinically observed progression and regression patterns of LV hypertrophy while requiring estimation of a limited number of parameters. Our model allows dynamic simulation of integrated cardiorenal function that covers time scales from second (cardiac cycle) to days/weeks (blood pressure changes and restoration of renal Na+ balance) to months (LV remodeling) with relatively low computational complexity. Complexity of the model does not go beyond ordinary differential equations, and simulating 6 months in model time can be completed in 3 min of real time. This model is adequate to describe the data considered, but further refinements may be needed to more fully represent the pathophysiological processes of hypertrophy and heart failure. The current model does not account for changes in cardiac tissue mechanical properties (e.g., stiffness) that likely occur due to cardiac fibrosis that accompanies hypertrophy. In addition, the model does not attempt to describe the biochemical processes by which mechanical signals are sensed and translated into cellular level changes in structure and function. Efforts to develop such models have recently been reviewed by Lee et al. (2016). The sympathetic nervous system plays an important but complex role in heart failure, but its representation in this model is limited, and its complex regulation has not been explored. The renal effects of ANP are also limited to effects on tubular sodium reabsorption, and vascular effects are not included. The roles of these important systems need to be more thoroughly investigated as we move toward a representative model of heart failure. Direct effects of neurohormonal systems on cardiac fibrosis and stiffness are also not considered. Our model does not incorporate aspects of changes in metabolism and oxygen utilization in the course of HF (Wende et al., 2017), which certainly play an important role in cardiac function and dysfunction. The current organ-system scale model may serve as a base model which may be further refined to incorporate these multi-scale processes.

## Data Availability Statement

The raw data supporting the conclusions of this article will be made available by the authors, without undue reservation.

## Author Contributions

KH wrote the manuscript, designed and performed the research, and analyzed the data. CV, SA, and SE designed and performed the research, analyzed the data, and revised the manuscript. All authors contributed to the article and approved the submitted version.

## Conflict of Interest

SE was an employee of Amgen, Inc. KH has received funding from AstraZeneca, Pfizer, Merck, and Takeda within the last 2 years. The authors declare that this study received funding from AstraZeneca, Pfizer, and Merck. The funder was not involved in the study design, collection, analysis, interpretation of data, writing of this article, or decision to submit it for publication. The remaining authors declare that the research was conducted in the absence of any commercial or financial relationships that could be construed as a potential conflict of interest.
