# Supplementary material for: Cardiorenal Systems Modeling: Left Ventricular Hypertrophy and Differential Effects of Antihypertensive Therapies on Hypertrophy Regression
Source: Front Physiol. 2021 Jun 16;12:679930. doi: 10.3389/fphys.2021.679930 (PMC8242213; doi:10.3389/fphys.2021.679930)
Supplement: Supplementary file 1 [file Table_1.pdf]

Supplementary Material

1 Full model equations

1.1 Renal vasculature

The glomeruli are modeled in parallel, and in series with the preafferent (interlobar, interlobular, and arcuate arterioles) and peritubular vasculature. Glomerular capillary resistance is assumed negligible. Thus, renal vascular resistance RVR is given by:

$$\mathrm{RVR} = \mathrm{R_{preaff}} + \frac{(R_{aa}+R_{ea})}{N_{nephrons}} + R_{peritubular} \quad \text{(S1)}$$

R_{preaff} and $R_{peritubular}$ are lumped resistances describing the total resistance of preafferent and peritubular vasculatures, respectively, while R_{aa} and R_{ea} are the resistances of a single afferent or efferent arteriole, as determined from Poiseuille's law, based on the arteriole's diameter d, length L, and blood viscosity μ:

$$R_{aa} = \frac{128\mu L_{aa}}{\pi d_{aa}^4}; \qquad R_{ea} = \frac{128\mu L_{ea}}{\pi d_{ea}^4} \quad \text{(S2)}$$

$N_{nephrons}$ is the number of nephrons. All nephrons are assumed identical, and the model does not account for spatial heterogeneity.

Renal blood flow (RBF) is a function of the pressure drop across the kidney and RVR, according to Ohm's law:

$$RBF = \frac{MAP - P_{renal-vein}}{RVR} + \frac{GFR\left(\frac{R_{ea}}{N_{nephrons}}\right)}{RVR} \quad \text{(S3)}$$

Renal venous pressure ($P_{renal\text{-}vein}$) is treated as constant. The second term in this equation accounts for lower flow through the efferent arterioles due to GFR. As an approximation, all filtrate is assumed reabsorbed back into the peritubular capillaries, so that peritubular flow is the same as afferent flow.

Glomerular capillary hydrostatic pressure P_{gc} is determined according to Ohm's law:

$$\mathrm{P_{gc}} = \mathrm{MAP} - \mathrm{RBF} * (\mathrm{R_{preaff}} + \mathrm{R_{aa}}/\mathrm{N_{nephrons}}) \quad \text{(S4)}$$

where P_{Bow} is pressure in the Bowman's space, and $\pi_{go\text{-}avg}$ is average glomerular capillary oncotic pressure and MAP is mean arterial pressure. These variables are described later.

Single nephron glomerular filtration rate (SNGFR) is defined according to Starling's equation, where K_f is the glomerular ultrafiltration coefficient

$$SNGFR = K_f\left(P_{gc} - P_{Bow} - \pi_{go-avg}\right) \quad \text{(S5)}$$

The total GFR is then the SNGFR multiplied by the number of nephrons:

$$GFR = SNGFR * N_{nephrons} \tag{S6}$$

1.2 Glucose filtration, reabsorption, and excretion

Glucose is filtered freely through the glomerulus, so that single nephron filtered glucose load is:

$$\Phi_{glu,filtered} = SNGFR * C_{glu} \tag{S7}$$

where C_{glu} is the plasma glucose concentration.

Glucose reabsorbed in the S1 and S2 segments of the proximal tubule is given by:

$$\Phi_{glu,reabs.s12} = \min(\Phi_{glu,filtered}, R_{glu,S12} * L_{pt,S12}) \tag{S8}$$

where $R_{glu,S12}$ is the rate of glucose reabsorption per unit length of the S1 and S2 segments together, and $L_{pt,S12}$ is the length of the PT S1 and S2 segments together. Similarly, glucose reabsorbed in the S3 segment is given by:

$$\Phi_{glu,reabs,S3} = \min(\ R_{glu,S3} * L_{pt,S3}, \Phi_{glu,filtered} - \Phi_{glu,reabs,S12}) \tag{S9}$$

Any glucose that is not reabsorbed then flows through the rest of the tubule and is ultimately excreted, so that the rate of urinary glucose excretion (R_{UGE}) is:

$$R_{UGE} = \Phi_{\mathrm{glu,out-PT}} = \Phi_{glu,filtered} - \Phi_{glu,reabs,S12} - \Phi_{glu,reabs,S3} \tag{S10}$$

Glucose reabsorption occurs exclusively in the PT through Na^+ glucose cotransporters (SGLT). SGLT2 in the S1 and S2 segments of the PT reabsorbs 90-97% of filtered glucose, while SGLT1 in the S3 segment reabsorbs the remaining 3-10%(1-5). At high plasma glucose concentrations, filtered glucose can exceed the kidney's capacity for reabsorption, and the excess glucose is excreted. $R_{glu,S12}$ and $R_{glu,s3}$ represent the number and function of SGLT2 and SGLT1 transporters respectively. The values were determined such that 95% of filtered glucose is reabsorbed in the S1 and S2 segments, while the remaining glucose was reabsorbed in the S3 segment, and so that all glucose is reabsorbed and urinary glucose excretion is zero for blood glucose concentrations up to 9 mmol/l(6).

1.3 Na^+ filtration and reabsorption in the PT

Similarly to glucose, Na^+ is freely filtered across the glomerulus, so that the single nephron filtered Na^+ load is given by:

$$\Phi_{\mathrm{Na,filtered}} = SNGFR * C_{Na} \tag{S11}$$

where C_{Na} is the plasma Na^+ concentration.

The rate of Na^+ reabsorption through SGLT2 equals the rate of glucose reabsorption in the S1 and S2 segments, since SGLT2 reabsorbs sodium and glucose at a 1:1 molar ratio:

$$\Phi_{\mathrm{Na,reabs-SGLT2}} = \Phi_{glu,reabs,S12} \tag{S12}$$

The rate of Na^+ reabsorption through SGLT1 is twice the rate of glucose reabsorption in the S3 segment, since SGLT1 reabsorb sodium and glucose at a 2:1 molar ratio:

$$\Phi_{\mathrm{Na,reabs-SGLT1}} = 2 * \Phi_{glu,reabs,S3} \tag{S13}$$

Total PT Na^+ reabsorption is then given by:

$$\Phi_{\mathrm{Na,reabs-PT}} = \Phi_{Na,filtered} * \left(\eta_{Na,reabs-PT,NHE3} + \eta_{Na,reabs-PT,other}\right) + \Phi_{\mathrm{Na,reabs-SGLT2}} + \Phi_{\mathrm{Na,reabs-SGLT1}} \tag{S14}$$

where $\eta_{\mathrm{Na,\ reabs-PT,NHE3}}$ and $\eta_{\mathrm{Na,reabs-PT,other}}$ are the fractional rates of PT sodium reabsorption through NHE3, and through mechanisms other than SGLT2 and NHE3. Na^+ flow rate out of the PT is then:

$$\Phi_{\mathrm{Na,out-PT}} = \Phi_{Na,filtered} - \Phi_{\mathrm{Na,reabs-PT}} \tag{S15}$$

For the remaining nephron segments, we approximate Na reabsorption in each segment as distributed uniformly along the length, and the rate of reabsorption per unit length is formulated so that the degree of flow-dependence can be varied. For a given segment, the nominal rate of reabsorption per unit length $r_{i,0}$ is given by the following, where η is the baseline fractional rate of reabsorption, $\Phi_{\mathrm{Na},0}(0)$ is the rate delivered to the segment under baseline conditions, and L is the segment length.

$$r_{i,0} = \frac{\eta_i \Phi_{\mathrm{Na,io}}(0)}{L_i} \tag{S16}$$

where i is the ascending loop of Henle (ALH), distal convoluted tubule (DCT), or connecting tubule and collecting duct (CNT/CD).

The actual rate per unit length r_i is then the nominal rate augmented by a flow-dependent component, as shown in Eq S17. The coefficient B determines the degree of flow-dependence: for B=0, there is no flow dependence; for B=1, changes in reabsorption are directly proportional to flow.

$$r_i = r_{i,0} + \frac{B_i \eta_i \left(\Phi_{\mathrm{Na,i}}(0) - \Phi_{\mathrm{Na,io}}(0)\right)}{L_i} \tag{S17}$$

Na flow along each segment is then:

$$\Phi_{Na,i}(\mathrm{x}) = \Phi_{\mathrm{Na,i}}(0) - \mathrm{r_i} x \tag{S18}$$

$\Phi_{\mathrm{Na,i}}(0)$ is obtained from the Na flow out of the preceding tubule segment.

1.4 Water reabsorption along the tubule

Water reabsorption in the PT is isosmotic. Therefore, water leaving the PT and entering the loop of Henle (LoH) is given by:

$$\Phi_{water,out-PT} = \Phi_{water,in-DCT} = SNGFR * \frac{\Phi_{osm,filtered}}{\Phi_{osm,out-PT}} \quad \text{(S19)}$$

where filtered osmolytes include both sodium and glucose:

$$\Phi_{osm,filtered} = 2 * \Phi_{Na,filtered} + \Phi_{glu,filtered} \quad \text{(S20)}$$

$$\Phi_{osm,out-PT} = 2 * \Phi_{Na,out-PT} + \Phi_{glu,out-PT} \quad \text{(S21)}$$

In the LoH, water is reabsorbed in the water permeable descending LoH (DLH) due to the osmotic gradient created by actively pumping sodium out of the water-impermeable ascending limb (ALH). The osmolality along the length of the DLH Osm_{DLH}, which is assumed in equilibrium with the osmolality in the surrounding interstitium Osm_{IS}, is given by:

$$Osm_{DLH}(x) = Osm_{IS}(x) = Osm_{DLH}(0)e^{\frac{r_{ALH}x}{\Phi_{water,in-DCT}Osm_{DLH}(0)}} \quad \text{(S22)}$$

Here, x is the distance along the tubule length, and R_{ALH} is the rate of sodium reabsorption per unit length in the ascending loop of Henle (Eq. A17). Water flow through the DLH is then given by:

$$\Phi_{water,DLH}(x) = \frac{\Phi_{water,DLH}(0)Osm_{DLH}(0)}{Osm_{DLH}(x)} \quad \text{(S23)}$$

The ALH and the DCT are modeled as impermeable to water, so that the flow through these segments equals the flow out of the DLH:

$$\Phi_{water,ALH}(x) = \Phi_{water,DCT}(x) = \Phi_{water,DLH}(L) \quad \text{(S24)}$$

In the collecting duct (CD), water reabsorption is driven by the osmotic gradient between the CD tubular fluid and the interstitium, and is modulated by vasopressin, as described later:

$$\Phi_{\text{water,reabs}-\text{CD}} = \mu_{vasopressin}\Phi_{\text{water,CD}}(0) * \left(1 - \frac{Osm_{CD}(L)}{Osm_{IS}(L)}\right) \quad \text{(S25)}$$

Where the osmolality in the CD $Osm_{CD}(L)$ accounts for sodium reabsorbed in the collecting duct:

$$Osm_{CD}(L) = \frac{\Phi_{\text{osm,cd}}(0) - 2*\left(\Phi_{\text{Na,cd}}(0) - \Phi_{\text{Na,cd}}(L)\right)}{\Phi_{\text{water,CD}}(0)} \quad \text{(S26)}$$

Then, single nephron water excretion rate is given by:

$$\Phi_{\text{water,CD}}(L) = \Phi_{\text{water,CD}}(0) - \Phi_{\text{water,reabs}-\text{CD}} \quad \text{(S27)}$$

And urine flow rate is then:

$$\Phi_{\text{urine}} = \text{SNGFR} * \Phi_{\text{water,CD}}(L) \quad \text{(S28)}$$

1.5 Total body sodium and water balance and peripheral sodium storage

A three-compartment model of volume homeostasis was used to describe total body sodium and water distribution. The compartments represent the blood, interstitium, and a peripheral sodium compartment in which sodium can be stored non-osmotically. Parameters for this portion of the model are given in Table S1. Sodium and water are assumed to move freely between the blood and interstitial fluid. Water and sodium intake rates were assumed constant. Then blood volume (V_b) and blood sodium (Na_{blood}) are the balance between intake and excretion of water and sodium respectively, and the intercompartmental transfer.

$$\frac{d}{dt}(V_b) = Water_{in} - Water_{out} + Q_{water}([Na]_{blood} - [Na]_{IF}) \quad \text{(S29)}$$

$$\frac{d}{dt}(Na_{blood}) = \Phi_{\text{Na,intake}} - \Phi_{\text{Na,excretion}} + Q_{Na}([Na]_{IF} - [Na]_{blood}) \quad \text{(S30)}$$

Sodium concentrations in the blood and interstitial compartments are assumed to equilibrate quickly. Change in interstitial fluid volume (IFV) is a function of intercompartmental water transfer.

$$\frac{d}{dt}(IFV) = Q_{water}([Na]_{IF} - [Na]_{blood}) \quad \text{(S31)}$$

When interstitial sodium concentration $[Na]_{IF}$ exceeds the normal equilibrium level $[Na]_{IF,ref}$, Na+ moves out of the interstitium and is sequestered in the peripheral Na+ compartment, at a rate of $\Phi_{Na,stored}$, where it is osmotically inactive. Thus, the change in the interstitial fluid sodium depends on intercompartmental transfer and peripheral storage. Sodium cannot be stored indefinitely, and thus there is a limit $Na_{stored,max}$ on how much sodium can be stored. The peripheral sodium compartment can be effectively removed from the model by setting $Q_{Na,stored}$ to zero.

$$\Phi_{\text{Na,stored}} = Q_{Na,stored} * \frac{(Na_{stored,max} - Na_{stored})}{Na_{stored,max}}([Na]_{JF} - [Na]_{IF,ref}) \quad \text{(S32)}$$

$$\frac{d}{dt}(Na_{stored}) = \Phi_{\text{Na,stored}} \quad \text{(S33)}$$

$$\frac{d}{dt}(Na_{IF}) = Q_{Na}([Na]_{blood} - [Na]_{IF}) - \Phi_{\text{Na,stored}} \quad \text{(S34)}$$

Blood and interstitial fluid sodium concentrations are then given by:

$$[Na]_{blood} = \frac{Na_{blood}}{V_B} \quad \text{(S35)}$$

$$[Na]_{IF} = \frac{Na_{IF}}{IFV} \quad \text{(S36)}$$

1.6 Tubular hydrostatic pressure.

Hydrostatic pressure in the Bowman's space is a key factor affecting GFR, and this pressure is influenced by both morphology and flow rates through the tubule. Changes in Na and water reabsorption along the nephron, which can occur either due to disease or treatments, can alter GFR by altering tubular pressures. Thus, dynamically modeling tubular pressures can be critical to understanding GFR changes.

Adapting from Jensen et al(7), tubular flow rates described in the main text can be used to determine tubular pressure. The change in intratubular pressure dP^* over a length of tubule dx can be defined according to Poiseuille's law as:

$$dP^* = -\frac{128\mu}{\pi D^4}\Phi_{water}(x)dx \tag{S37}$$

Eq. S38 describes the relationship between transtubular pressure P and tubular diameter D, where D_c is the diameter at control pressure P_c, and β is the exponent of tubular distensibility.

$$\frac{D}{D_c} = \left(\frac{P}{P_c}\right)^{\beta} \tag{S38}$$

Substituting and assuming uniform interstitial pressure throughout the kidney, we obtain:

$$dP = -\frac{128\eta}{\pi D_c^4}\left(\frac{P_c}{P}\right)^{4\beta}\Phi_{water}(x)\,dx \tag{S39}$$

Integrating over a tubule segment length, we obtain inlet pressure as a function of the outlet pressure and the flow rate:

$$P_{in} = \left[P_{out}^{4\beta+1} + \frac{(4\beta+1)128\eta P_c^{4\beta}}{\pi D_c^4}\int_0^L \Phi_{water}(x)dx\right]^{\frac{1}{4\beta+1}} \tag{S40}$$

The pressure calculated at the inlet to the PT is used as P_{Bow} in Eq. S5 above.

Because the diameter of the CNT/CD changes as nephrons coalescence, calculating pressure along this segment is challenging. Under normal conditions, pressure drops 5-7mmHg across the CNT/CD. Thus, an effective control diameter was calculated to give this degree of pressure drop under baseline conditions.

1.7 Glomerular capillary oncotic pressure

The glomerular capillary oncotic pressure is calculated using the Landis Pappenheimer equation(8), where C_{prot} is the concentration of protein at the point of interest.

$$\pi = 1.629 * C_{prot} + 0.2935 * C_{prot}^2 \tag{S41}$$

Plasma protein ($C_{prot-plasma}$) is assumed constant. Protein concentration at the distal end of the glomerulus ($C_{prot-glom-out}$) is determined as:

$$C_{prot-glom-out} = C_{prot-plasma} * \frac{RBF}{RBF-GFR} \tag{S42}$$

Protein concentration is assumed to be varying linearly along the capillary length, and thus the oncotic pressure π_{go-avg} is calculated using the average of the plasma protein concentration and protein concentration at the distal end of the glomerulus.

.

1.8 Regulatory mechanisms

Multiple control mechanisms act on the system to allow simultaneous control of C_{NA}, CO, MAP, glomerular pressure, and RBF. For each control mechanism, the feedback signal μ is modeled by one of two functional forms. The choice of functional form is determined by whether a steady state error is allowed in the controlled variable X. When a steady state error is not allowed (i.e. X always eventually returns to the setpoint X_0), the effect is defined by a proportional-integral (PI) controller. The initial feedback signal is proportional to the magnitude of the error ($X-X_0$), with gain G. But the feedback continues to grow over time as long as any error exists, until the error returns to zero. The integral gain K_i determines the speed of return to steady-state.

$$\mu = 1 + G * ((X - X_0) + K_i * \int (X - X_0)dt) \qquad \text{(S43)}$$

All other mechanisms, for which the controlled variable can deviate from the setpoint at steady-state, are described by a logistic equation that produces a saturating response characteristic of biological signals:

$$\mu = 1 + S * \left(\frac{1}{1+\exp\left(\frac{X-X_0}{m}\right)} - 0.5\right) \qquad \text{(S44)}$$

Here, m defines the slope of the response around the operating point, and S is the maximal response as X goes to $\pm\infty$.

1.8.1 Control of plasma Na concentration by vasopressin

Changes in plasma osmolality are sensed via osmoreceptors, stimulating vasopressin secretion, which exerts control of water reabsorption in the CNT/CD. To ensure that blood sodium concentration C_{Na} is maintained at its setpoint $C_{Na,0}$ at steady state, this process is modeled by a PI controller:

$$\mu_{vasopressin} = 1 + G_{Na-vp} * \left(C_{Na} + K_{i-vp} * \int \left(C_{Na} - C_{Na,0}\right)dt\right) \qquad \text{(S45)}$$

The parameters $G_{Na\text{-}vp}$ and $K_{i\text{-}vp}$ are gains of proportional and integral control, respectively.

1.8.2 Tubular pressure natriuresis

For homeostasis, Na excretion over the long-term must exactly match Na intake (the principle of Na balance). Any steady-state Na imbalance would lead to continuous volume retention or loss – an untenable situation. Pressure-natriuresis(9), wherein changes in renal perfusion pressure (RPP) induce changes in Na excretion, insures that Na balance is maintained. It may be partially achieved through neurohumoral mechanisms including the RAAS, but there is also an intrinsic pressure-mediated effect on tubular Na reabsorption, where renal interstitial hydrostatic pressure (RIHP) is believed to be the driving signal. RIHP is a function of peritubular capillary pressure, and is calculated according to Ohm's law:

$$P_{peritubular} = MAP - RBF * \left(R_{preaff} + \frac{R_{aff}+R_{eff}}{N_{nephrons}}\right) \quad (S46)$$

As a simplification, we assume an increase in peritubular pressure will generate a proportional increase in RIHP. Since the kidney is encapsulated, we assume interstitial pressure equilibrates and changes in one region are transduced across the kidney. The relationship between RIHP and fractional Na reabsorption rate of each tubular segment is then modeled as:

$$\eta_{i-sodreab} = \eta_{i-sodreab,0} * \left(1 + S_{P-N,i} * \left(\frac{1}{1+\exp(RIHP-RIHP_0)} - 0.5\right)\right) \quad (S47)$$

where i = PT, LoH, DCT, or CNT/CD. $\eta_{i-sodreab,0}$ is the nominal fractional rate of reabsorption for that tubule segment. $RIHP_0$ defines the setpoint pressure and is determined from RIHP at baseline for normal Na intake. $S_{P-N,i}$ defines the maximal signal as RIHP goes to ∞.

1.8.3 Control of macula densa sodium concentration by tubuloglomerular feedback

Tubuloglomerular feedback (TGF) helps stabilize tubular flow by sensing Na concentration in the macula densa, which sits between the LoH and DCT, and providing a feedback signal to inversely change afferent arteriole diameter. The TGF effect is defined as:

$$\mu_{TGF} = 1 + S_{TGF} * \left(\frac{1}{1+\exp\left(\frac{C_{Na,MD,0}-C_{Na,MD}}{m_{TGF}}\right)} - 0.5\right) \quad (S48)$$

The basal afferent arteriole resistance Raa is then multiplied by μ_{TGF} to obtain the ambient afferent arteriolar resistance. The setpoint $C_{Na,MD,0}$ is the Na concentration out of the LoH and into the DCT in the baseline state at normal Na intake.

1.8.4 Myogenic autoregulation of glomerular pressure

Glomerular hydrostatic pressure is normally tightly autoregulated, and changes very little in response to large changes in blood pressure. This autoregulation is in part through myogenic autoregulation of the preglomerular arterioles. While the pressure drop and thus myogenic response varies along the arteriole length, we make the simplifying assumption that the preafferent vasculature responds to control pressure at the distal end.

$$\mu_{autoreg} = 1 + S_{autoreg} * \left(\frac{1}{1+\exp\left(\frac{P_{preafferent}-P_{preafferent,0}}{m_{autoreg}}\right)} - 0.5\right) \quad (S49)$$

Pressure at the distal end of the preafferent vasculature is given by:

$$P_{preafferent} = MAP - RBF * R_{preaff} \quad (S50)$$

The basal preafferent arteriole resistance R_{preaff} is then multiplied by $\mu_{autoreg}$ to obtain the ambient preafferent arteriolar resistance.

1.8.5 Control of cardiac output

CO, which describes total blood flow to body tissues, tends to return normal values following a perturbation, as it is controlled through multiple feedback mechanisms (10). CO regulation is a complex phenomenon that occurs over multiple time scales, but we focus only on long-term control (days to weeks), which is thought to be achieved through whole-body autoregulation - the intrinsic ability of organs to adjust their resistance to maintain constant flow(10). The total effect of local autoregulation of all organs is that TPR is adjusted to maintain CO at a constant resting level. The feedback between CO and TPR is modeled with a PI controller, such that CO is controlled to its steady-state setpoint CO_0.

$$\mathrm{TPR} = \mathrm{TPR}_0 * \left(1 + \mathrm{G}_{\mathrm{CO-tpr}} * \left(\mathrm{CO} + \mathrm{K}_{\mathrm{i-tpr}} * \int(\mathrm{CO} - \mathrm{CO}_0)\mathrm{dt}\right)\right) \qquad \text{(S51)}$$

1.8.6 Renin-Angiotensin-Aldosterone System submodel

Renin is secreted at a nominal rate $SEC_{ren,0}$ modulated by macula densa sodium flow, renal sympathetic nerve activity (described later), as well as by a strong negative feedback from Angiotensin II (AngII) bound to the AT1 receptor.

$$SEC_{renin} = \mu_{md-renin} * \mu_{rsna} * \mu_{AT1} * SEC_{renin,0} \qquad \text{(S52)}$$

The macula densa releases renin in response to reduced sodium flow:

$$\mu_{md-renin} = e^{-A_{md-ren}(\phi_{Na,md} - \phi_{Na,md,0})} \qquad \text{(S53)}$$

We have found that the inhibitory effect of AT1-bound AngII on renin secretion can be well described by the following relationship:

$$\mu_{AT1} = \mathrm{A}_{\mathrm{AT1,ren}} \left(\frac{\mathrm{AT1-bound-AngII}}{\mathrm{AT1-bound-AngII}_0}\right) \qquad \text{(S54)}$$

Plasma renin concentration (PRC) is then given by:

$$\frac{d(PRC)}{dt} = SEC_{renin} - K_{d,renin} * PRC \qquad \text{(S55)}$$

Where $K_{d,renin}$ is the renin degradation rate. Plasma renin activity (PRA) can be related to PRC by the conversion factor 0.06 (ng/ml/hr)/(pg/ml).

Angiotensin I (AngI) is formed by PRA, assuming that its precursor angiotensinogen is available in excess and the plasma renin activity (PRA) is the rate-limiting step. AngI is then converted to AngII by the enzymes ACE and chymase, and is degraded at a rate of $K_{d,AngI}$.

$$\frac{d(AngI)}{dt} = PRA - (ACE + Chymase) * AngI - K_{d,AngI}AngI \qquad \text{(S56)}$$

Angiotensin II is formed from the action of ACE and chymase on AngI, it can be eliminated by binding to either the AT1 or AT2 receptors at the rate K_{AT1} and K_{AT2} respectively, and is degraded at a rate of $K_{d,AngII}$.

$$\frac{d(AngII)}{dt} = (ACE + \ Chymase) * AngI - (K_{AT1} + K_{AT2}) * AngII - K_{d,AngII}AngII \quad \text{(S57)}$$

The complex of Angiotensin II bound to the AT1 receptor is the physiologically active entity within the pathway, and is given by:

$$\frac{d\left(AT1_{bound_{AngII}}\right)}{dt} = (K_{AT1}) * AngII - K_{d,AT1}AT1\_bound\_AngII \quad \text{(S58)}$$

AT1-bound AngII has multiple physiologic effects, including constriction of the efferent, as well and preglomerular, afferent, and systemic vasculature, sodium retention in the PT, and aldosterone secretion. Each effect is modeled as:

$$\mu_{AT1,i} = 1 + S_{AT1,i} * \left(\frac{1}{1+\exp\left(\frac{AT1-bound_{AngII_0} - AT1-bound_{AngII}}{m_{AT1,i}}\right)} - 0.5 \right) \quad \text{(S59)}$$

where i represents the effect on efferent, afferent, preafferent, or systemic resistance, PT sodium reabsorption, or aldosterone secretion.

Aldosterone is the second physiologically active entity in the RAAS pathway, acting by binding to mineralocorticoid receptors (MR) in the CNT/CD and DCT to stimulate sodium reabsorption. MR-bound aldosterone is modeled as the nominal concentration Aldo,0 modulated by the effect of AT1-bound AngII, and the normalized availability of MR receptors (1 in the absence of an MR antagonist).

$$MR - bound\_Aldo = Aldo_0 * \mu_{AT1} * MR \quad \text{(S60)}$$

The effects of MR-bound aldosterone on CNT/CD and DCT sodium reabsorption are modeled as:

$$\mu_{aldo,i} = 1 + S_{aldo,i} * \left(\frac{1}{1+exp\left(\frac{MR-bound\ Aldo_0 - MR-bound\ Aldo}{m_{aldo,i}}\right)} - 0.5 \right) \quad \text{(S61)}$$

Where i is the CNT/CD or DCT.

1.8.7 Atrial Natriuretic Peptide

Atrial Natriuretic Peptide (ANP) is increased in response to increased in left ventricle end diastolic pressure (LVEDP) above normal levels

$$ANP = ANP_0 \, e^{max(LV\ EDP - 10mmHg, 0) * m_{edp-anp}} \quad \text{(S62)}$$

Here, ANP0 is the normal ANP level (~ 50 pg/ml), and medp-anp is a fitting constant obtained by fitting the observed LV EDP and ANP data in Maeda 1998 (11).

The effect of ANP on CNT/CD sodium reabsorption is modeled as:

$$\mu_{anp,cnt-cd} = 1 - S_{anp,cnt-cd} * \left(\frac{1}{1+exp\left(\frac{1-\frac{ANP}{ANP0}}{m_{ANP}}\right)} - 0.5 \right) \tag{S63}$$

1.8.8 Renal Sympathetic Nerve Activity

We do not attempt to model the complexities of dynamics RSNA regulation. However, the effects of a sustained change in RSNA on renal vascular resistance and renin secretion are modeled. The effect of RSNA on renin secretion is modeled as:

$$\mu_{rsna-renin} = 1 + m_{rsna}\left(\frac{RSNA}{RSNA0} - 1\right) \tag{S64}$$

The effect of RSNA on preafferent arteriole resistance is modeled as:

$$\mu_{rsna-preaff} = 1 + S_{rsna-preaff} * \left(\frac{1}{1+exp\left(\frac{1-\frac{RSNA}{RSNA0}}{m_{RSNA}}\right)} - 0.5 \right) \tag{S65}$$

1.9 Cardiac mechanics

The ventricular mechanics portion of the model was adapted from a previously published model by Arts, Bovendeerd, and colleagues (12, 13). Many equations were used verbatim from these previous papers. We repeat those equations here for the reader's convenience, but refer the reader to the original publication for more complete explanation. Here we present equations for the left ventricle; analogous equations were used for the right ventricle.

The volume of blood inside the left ventricle chamber V_{lv} is given by:

$$\frac{d(V_{lv})}{dt} = Q_{mitral} - Q_{aorta} \tag{S66}$$

where Q_{mitral} and Q_{aorta} are blood flow rates through the mitral and aortic valves, respectively, as described later. Bovendeerd et al showed that left ventricular pressure P_{lv} can be related to LV volume V_{lv} and LV wall volume V_w by the following (Ref 6, Eq. 7):

$$P_{lv} = \frac{1}{3}\left(\sigma_f - 2\sigma_{m,r}\right)\ln\left(1 + \frac{V_w}{V_{lv}}\right) \tag{S67}$$

Here σ_f and $\sigma_{m,r}$ are mechanical stresses in the myocardium along the longitudinal fiber and the radial direction respectively. σ_f is comprised of the sum of the passive stress along the fiber $\sigma_{m,f}$ and active fiber stress σ_a. The passive stress along the fiber is a function of the longitudinal stretch along the fiber λ_f and the myocardial longitudinal stiffness c_f (Ref 6, Eq. 14).

$$\sigma_{m,f}(\lambda_f) = \begin{Bmatrix} \sigma_{f0}\left(e^{c_f(\lambda_f - 1)} - 1\right) \\ 0 \end{Bmatrix} \begin{matrix} \lambda_f \geq 1 \\ \lambda_f < 1 \end{matrix} \tag{S68}$$

Mean passive radial stress is a function of the radial stretch λ_r and the myocardial radial stiffness c_r (Ref 6, Eq. 15).

$$\sigma_{m,r}(\lambda_r) = \begin{Bmatrix} \sigma_{r0}\left(e^{c_r(\lambda_r - 1)} - 1\right) \\ 0 \end{Bmatrix} \begin{matrix} \lambda_r \geq 1 \\ \lambda_r < 1 \end{matrix} \tag{S69}$$

As shown by Bovendeerd et al, the longitudinal stretch λ_f is related to chamber blood volume and wall volume by (Ref 6, Eq. 8):

$$\lambda_f = \left(\frac{V_{lv} + \frac{1}{3}V_w}{V_{lv,cavity} + \frac{1}{3}V_w}\right)^{\frac{1}{3}} \tag{S70}$$

$V_{lv,cavity}$ is the chamber volume at zero transmural pressure.

The radial stretch λ_r is given by (Ref 6, Eq. 9):

$$\lambda_r = \lambda_f^{-2} \tag{S71}$$

where C_f and C_r are the stiffness of the myocardial tissue in the longitudinal and radial directions, respectively.

LV active stress is a function of contractility (c), sarcomere length l_s, sarcomere shortening velocity V_s, and time elapsed since beginning of contraction (t_a). These equations were taken exactly as shown in Ref 6, Eq. 10 - 13.

2 Supplementary Tables and Figures

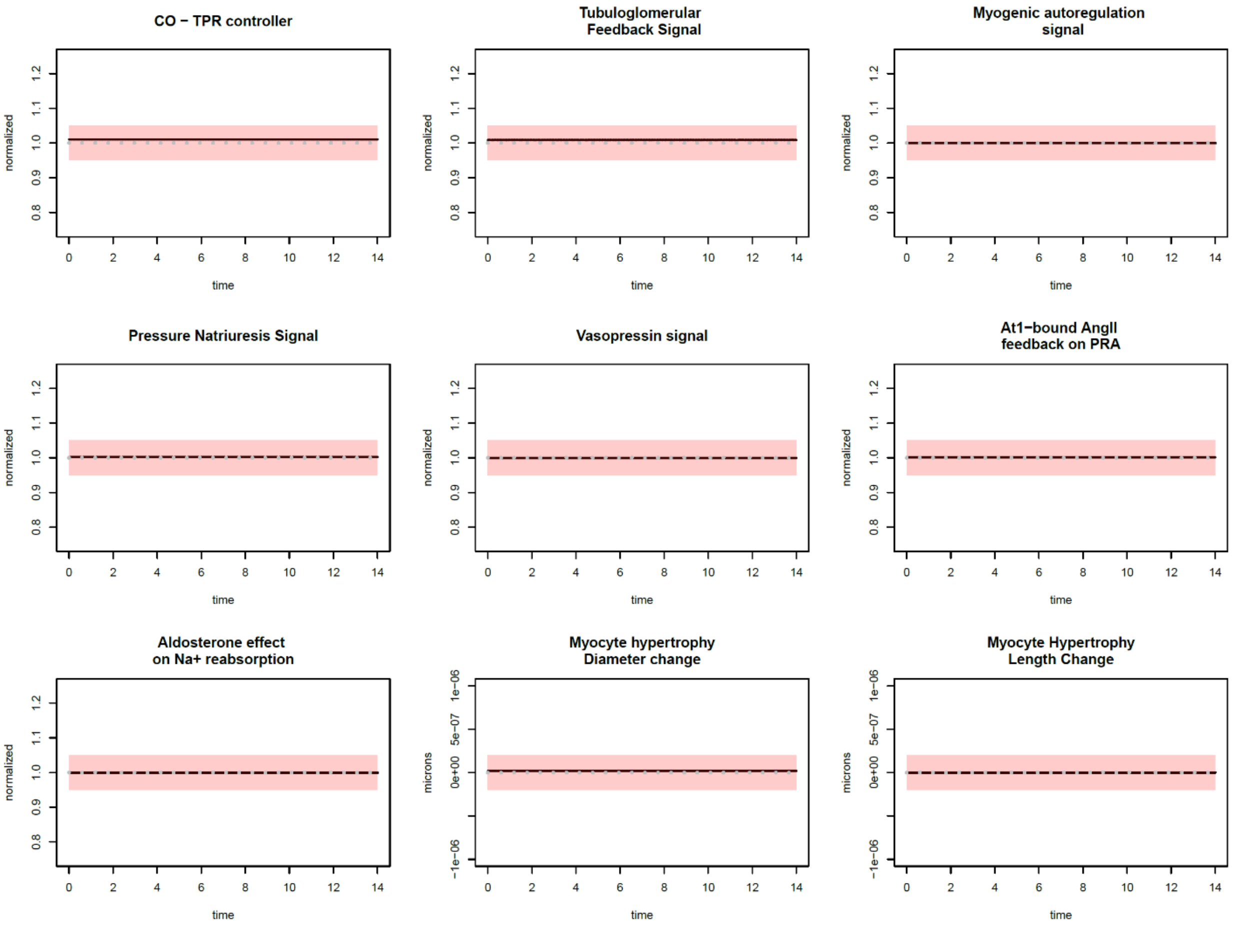

Supplementary Figure 1. At baseline, feedback mechanisms in the model are stable and at their setpoint value. Myocyte diameter and length are also at their baseline value and are unchanging, indicating that LV peak systolic stress and LV end diastolic stress are at or below threshold levels for remodeling.

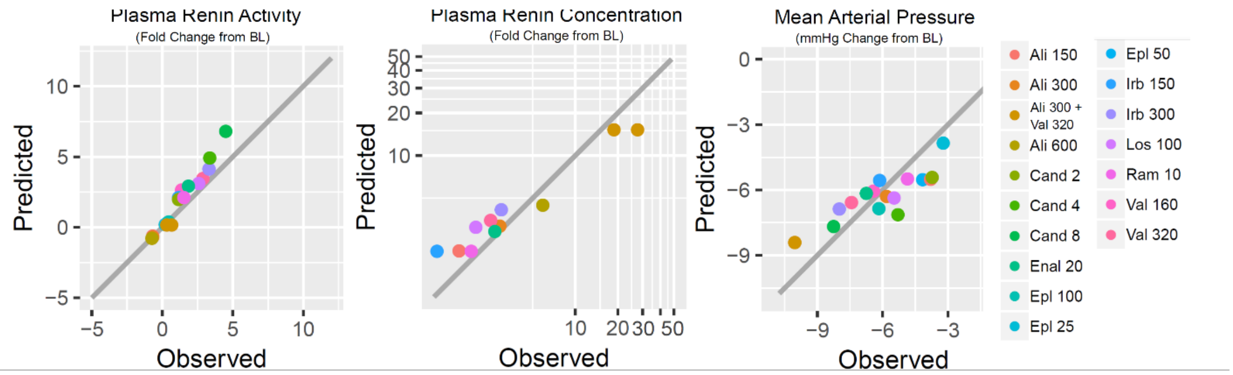

Supplementary Figure 2. Validation test for ability of model to simulation response to antihypertensive therapies. Effects of antihypertensive therapies (aliskiren [Ali], valsartan [Val], candesartan [cand], enalapril [enal], eplerenone [epl], irbesartan [irb], losartan [los], ramapril [ram] were previously calibrated and validated in the renal-only version of this model(14). As validation of the integrated model, we repeated these simulations, and show here that the integrated model

produces changes in plasma renin activity, plasma renin concentration, and mean arterial pressure consistent with clinically observed levels (see (14) for full description of simulation and data used.

Supplementary Table 1. Additional Cardiac Model Parameters

Parameter	Definition	Value	Units
C	cardiac contractility	1	-
c_f	LV stiffness along the fiber	12	-
c_r	LV radial stiffness	9	-
$C_{f,RV}$	RV stiffness along the fiber	9	-
$C_{r,RV}$	RV radial stiffness	9	-
$l_{s,a0}$	Sarcomere length below which active stress becomes zero	1.9	μm
$l_{s,ar}$	Sarcomere length to which the reference stress σ_{ar} is referenced to	2	μm
σ_{ar}	Active stress scaling constant	55	kPa
σ_{f0}	Longitudinal passive stress at zero stretch	0.9	kPa
σ_{r0}	Radial passive stress at zero stretch	0.2	kPa
v_0	Unloaded sarcomere shortening velocity	50	μm/s
$V_{w,rv}$	RV wall volume	100	mL

Supplementary Table 2. Circulatory Model Parameters

Parameter	Definition	Value	Units

R_{art0}	arterial resistance	5e6	Pa-s/m^3
R_{per0}	peripheral vascular resistance	1.27e8	Pa-s/m^3
R_{ven}	venous resistance	5e6	Pa-s/m^3
R_{mitral}	Mitral valve resistance	1e6	Pa-s/m^3
$R_{pulm,art}$	pulmonary arterial resistance	3e6	Pa-s/m^3
$R_{pulm,ven}$	pulmonary venous resistance	6.4e6	Pa-s/m^3
R_{RA}	right atrial resistance	1e6	Pa-s/m^3
V_{art0}	arterial volume at zero transmural pressure	450	mL
V_{per0}	peripheral volume at zero transmural pressure	420	mL
V_{ven0}	venous volume at zero transmural pressure	3000	mL
$V_{pulm,art0}$	pulmonary arterial volume at zero transmural pressure	40	mL
$V_{pulm,ven0}$	pulmonary venous volume at zero transmural pressure	250	mL
V_{RV0}	right ventricle volume at zero transmural pressure	75	mL
$V_{w,RV}$	right ventricle wall volume	100	mL
C_{art}	arterial compliance	1.1e-8	m^3/Pa
C_{per}	peripheral vascular compliance	1e-8	m^3/Pa
C_{ven}	venous compliance	1.8e-7	m^3/Pa
$C_{art,pulm}$	arterial compliance	2e-8	m^3/Pa

$C_{per,ven}$	peripheral vascular compliance	1.65e-7	m^3/Pa
I_{art}	arterial **inertance**	60	$kPa-s/m^3$
I_{pulm}	pulmonary arterial **inertance**	60	$kPa-s/m^3$

Supplementary Table 3. Renal Model Parameters

Parameter	Definition	Value	Units
β	Tubular compliance	0.2	-
$\eta_{Na, CNT-CD}$	Fractional rate of CNT/CD Na+ reabsorption	0.827*	-
$\eta_{Na, DCT}$	Fractional rate of DCT Na+ reabsorption	0.5	-
$\eta_{Na, ALH}$	Fractional rate of PT Na+ reabsorption through PT NHE3	0.8	-
$\eta_{Na, reabs-PT,NHE3}$	Fractional rate of PT Na+ reabsorption through PT NHE3	0.3	-
$\eta_{Na, reabs-PT,other}$	Fractional rate of PT Na+ reabsorption through non-NHE3, non-SGLT2 mechanisms	0.35	-
$\Phi_{Na,ALH0}$	Rate of sodium delivered to the ALH under baseline conditions	2.02*	µl/min
$\Phi_{Na,intake}$	Sodium intake rate	100	mEq/day
B	LoH flow dependence coefficient	0.75	-

C_{glu}	Plasma glucose concentration	5	mmol/L
C_{prot}	Plasma protein concentration	7	g/dl
d_{aa0}	Nominal afferent arteriole diameter	11	µm
d_{ea0}	Nominal efferent arterial diameter	16.5	µm
$D_{c,cnt-cd}$	Connecting tubule/collecting duct effective diameter at control pressure	22	µm
$D_{c,dct}$	Distal convoluted tubule diameter at control pressure	17	µm
$D_{c,lh}$	Loop of Henle diameter at control pressure	17	µm
$D_{c,pt}$	Proximal tubule diameter at control pressure	27	µm
Kf	Glomerular ultrafiltration coefficient	4	L/min-mmHg
L_{aa}	Average afferent arteriole length	73.6*	µm
L_{ea}	Average efferent arteriole length	73.6*	µm
L_{CNT-CD}	Connecting tubule/collecting duct effective length	10	mm
L_{dct}	Distal convoluted tubule length	5	mm
$L_{LoH,Asc}$	Ascending loop of Henle length	10	mm
$L_{LoH,Desc}$	Descending loop of Henle length	10	mm
$L_{pt,s1}$	Length of the PT S1 segment	5	mm
$L_{pt,s2}$	Length of the PT S2 segment	5	mm
$L_{pt,s3}$	Length of the PT S3 segment	4	mm

$N_{nephrons}$	Number of nephrons	2e6	-
$[Na]_{ref}$	Normal blood/IF equilibrium sodium concentration	140	mmol/L
Na_{stored}	Maximum peripherally stored sodium	2000	Mmol
$P_{c,cnt\text{-}cd}$	CNT/CD control pressure	5	mmHg
$P_{c,dt}$	DCT control pressure	6	mmHg
$P_{c,lh,asc}$	Ascending loop of Henle control pressure	7	mmHg
$P_{c,lh,desc}$	Descending loop of Henle control pressure	8	mmHg
$P_{c,pt,s1}$	PT S1 segment control pressure	20.2	mmHg
$P_{c,pt,s2}$	PT S2 control pressure	15	mmHg
$P_{c,pt,s3}$	PT S3 control pressure	11	mmHg
Q_{water}	Rate constant for water transfer between blood and IF	1	1/min
Q_{Na}	Rate constant for sodium transfer between blood and IF	1	1/min
$Q_{Na,storage}$	Rate constant for sodium storage/release from the peripheral compartment	0.1	1/min
$r_{glu,s12}$	Rate of glucose reabsorption through SGLT2 in the PT S1 and S2 segment per unit length	0.2	mmol/min/mm
$r_{glu,s3}$	Rate of glucose reabsorption through SGLT1 in the PT S3 segment per unit length	0.025	mmol/min/mm
$R_{preaff,0}$	Nominal preafferent arteriole resistance	14	mmHg-min/L

μ	Blood viscosity	5e-7	mmHg-min
$Water_{in}$	Water intake rate	2.1	L/day

*Value calculated based on other parameters under baseline conditions

Supplementary Table 4. Regulatory mechanisms model parameters

Parameter	Definition	Value	Units
$\Phi_{Na,md,0}$	Setpoint for sodium flow delivered to the macula densa	0.885*	μl/min
$A_{md\text{-}renin}$	Scaling factor for macula densa renin secretion	0.9	-
Aldo`	Aldosterone concentration setpoint	85	pg/ml
$AT1\text{-}bound\text{-}AngII_0$	AT1-bound AngII setpoint	16.6*	pg/ml
$C_{NA,MD,0}$	Macula Densa sodium concentration setpoint	63.3*	mEq/L
$G_{CO\text{-}tpr}$	Proportional gain for cardiac output – TPR controller	0.1	-
$G_{Na\text{-}vp}$	Proportional gain for vasopressin control of sodium concentration	0.1	-
$K_{i\text{-}tpr}$	Integral gain for vasopressin control of sodium concentration	0.1	-
$K_{i\text{-}vp}$	Integral gain for vasopressin control of sodium concentration	0.005	-
$m_{aldo,cnt\text{-}cd}$	Slope factor for aldosterone effect on CNT/CD sodium reabsorption	0.5	-
$m_{aldo,dct}$	Slope factor for aldosterone effect on CNT/CD sodium reabsorption	0.5	-

$m_{AT1\text{-}aff}$	Slope factor for AT1-bound AngII effect on afferent resistance	16	-
$m_{AT1\text{-}eff}$	Slope factor for AT1-bound AngII effect on efferent resistance	16	-
$m_{AT1\text{-}preaff}$	Slope factor for AT1-bound AngII effect on preafferent resistance	16	-
$m_{AT1\text{-}pt}$	Slope factor for AT1-bound AngII effect on proximal tubule Na+ reabsorption	16	-
$m_{autoreg}$	Myogenic autoregulation slope factor	2	-
m_{TGF}	Tubuloglomerular feedback signal slope factor	6	-
$P_{preafferent,0}$	Preafferent arteriole pressure setpoint	71*	mmHg
$RIHP_0$	Renal interstitial hydrostatic pressure setpoint	9.66*	mmHg
$S_{aldo\text{-}cnt\text{-}cd}$	Scaling factor for aldosterone effect on CNT/CD sodium reabsorption	0.2	-
$S_{aldo\text{-}dct}$	Scaling factor for aldosterone effect on DCT sodium reabsorption	0.05	-
$S_{AT1\text{-}aldo}$	Scaling factor for AT1-bound AngII effect on aldosterone secretion	0.02	-
$S_{AT1\text{-}aff}$	Scaling factor for AT1-bound AngII effect on afferent resistance	0.8	-
$S_{AT1\text{-}eff}$	Scaling factor for AT1-bound AngII effect on efferent resistance	0.8	-
$S_{AT1\text{-}preaff}$	Scaling factor for AT1-bound AngII effect on preafferent resistance	0.8	-

$S_{AT1\text{-}pt}$	Scaling factor for AT1-bound AngII effect on proximal tubule Na+ reabsorption	0.1	-
$S_{autoreg}$	Preafferent autoregulation signal scaling factor	1	-
S_{TGF}	Tubuloglomerular feedback signal scaling factor	0.7	-
$S_{P\text{-}N,CNT\text{-}DC}$	CNT-DC pressure-natriuresis signal scaling factor	0.5	-
$S_{P\text{-}N,DCT}$	DCT pressure-natriuresis signal scaling factor	0.1	-
$S_{P\text{-}N,LoH}$	LoH pressure-natriuresis signal scaling factor	0.1	-
$S_{P\text{-}N,PT}$	PT pressure-natriuresis signal scaling factor	0.5	-

Supplementary Table 5. Renin Angiotensin Aldosterone System model parameters

Parameter	Definition	Value	Units
ACE	ACE activity	47.65*	/min
Chymase	Chymase activity	2.5*	/min
K_{AT1}	AT1-receptor binding rate	12.1*	/min
K_{AT2}	AT2-receptor binding rate	4*	/min
$K_{d,AngI}$	AngI degradation rate	0.0924	/min
$K_{d,AngI}$	AngII degradation rate	0.146	/min
$K_{d,AT1}$	AT1-bound AngII degradation rate	3.47	/min

$K_{d,renin}$	Renin degradation rate	4	/min

Supplementary Table 6. Model Initial Conditions

Variable	Definition	Value	Units
AngI	Angiotensin I	8.164*	pg/mg
AngII	Angiotensin II	5.17*	pg/mg
AT1-bound AngII	AT1-bound AngII	16.6*	pg/mg
AT2-bound AngII	AT2-bound AngII	5.5*	pg/mg
CO	Cardiac Output	5	L/min
IFV	Interstitial Fluid Volume	12	L
Na_{blood}	Blood sodium amount	700	mEq
Na_{IF}	Interstitial sodium amount	2100	mEq
Na_{stored}	Stored sodium amount	0	mEq
PRC	Plasma Renin Concentration	17.84	pg/ml
V_{art}	Arterial volume	573*	mL
V_b	Blood Volume	5	L
V_{LV}	LV chamber volume	110	mL
V_{RV}	RV chamber volume	182*	mL
V_{per}	Peripheral circulation volume	441*	mL

$V_{pulm,art}$	Pulmonary arterial volume	123*	mL
$V_{pulm,ven}$	Pulmonary venous volume	476*	mL
V_{venous}	Venous volume	3278*	mL

1. Vallon V, Platt KA, Cunard R, Schroth J, Whaley J, Thomson SC, et al. SGLT2 Mediates Glucose Reabsorption in the Early Proximal Tubule. J Am Soc Nephrol. 2011;22(1):104-12.
2. Vallon V. The proximal tubule in the pathophysiology of the diabetic kidney. American Journal of Physiology-Regulatory Integrative and Comparative Physiology. 2011;300(5):R1009-R22.
3. Novikov A, Vallon V. Sodium glucose cotransporter 2 inhibition in the diabetic kidney: an update. Curr Opin Nephrol Hy. 2016;25(1):50-8.
4. Gorboulev V, Schurmann A, Vallon V, Kipp H, Jaschke A, Klessen D, et al. Na+-D-glucose Cotransporter SGLT1 is Pivotal for Intestinal Glucose-Absorption and Glucose-Dependent Incretin Secretion. Diabetes. 2012;61(1):187-96.
5. Rieg T, Masuda T, Gerasimova M, Mayoux E, Platt K, Powell DR, et al. Increase in SGLT1-mediated transport explains renal glucose reabsorption during genetic and pharmacological SGLT2 inhibition in euglycemia. Am J Physiol-Renal. 2014;306(2):F188-F93.
6. DeFronzo RA, Hompesch M, Kasichayanula S, Liu X, Hong Y, Pfister M, et al. Characterization of renal glucose reabsorption in response to dapagliflozin in healthy subjects and subjects with type 2 diabetes. Diabetes Care. 2013;36(10):3169-76.
7. Jensen PK, Christensen O, Steven K. A mathematical model of fluid transport in the kidney. Acta physiologica Scandinavica. 1981;112(4):373-85.
8. Landis EM. Exchange of substances through the capillary walls. Handbook of Physiology, Circulation II. 1963:961-1034.
9. Guyton AC, Coleman TG, Cowley AW, Manning RD, Norman RA, Ferguson JD. A systems analysis approach to understanding long-range arterial blood pressure control and hypertension. Circulation research. 1974;35(2):159-76.
10. Coleman TG, Granger HJ, Guyton AC. Whole-body circulatory autoregulation and hypertension. Circulation research. 1971;28(5):Suppl 2:76-87.
11. Maeda K, Tsutamoto T, Wada A, Hisanaga T, Kinoshita M. Plasma brain natriuretic peptide as a biochemical marker of high left ventricular end-diastolic pressure in patients with symptomatic left ventricular dysfunction. Am Heart J. 1998;135(5 Pt 1):825-32.
12. Arts T, Delhaas T, Bovendeerd P, Verbeek X, Prinzen FW. Adaptation to mechanical load determines shape and properties of heart and circulation: the CircAdapt model. American Journal of Physiology - Heart and Circulatory Physiology. 2005;288(4):H1943-H54.
13. Bovendeerd PH, Borsje P, Arts T, van DV. Dependence of intramyocardial pressure and coronary flow on ventricular loading and contractility: a model study. AnnBiomedEng. 2006;34(12):1833-45.
14. Hallow KM, Lo A, Beh J, Rodrigo M, Ermakov S, Friedman S, et al. A model-based approach to investigating the pathophysiological mechanisms of hypertension and response to antihypertensive therapies: extending the Guyton model. American journal of physiology Regulatory, integrative and comparative physiology. 2014;306(9):R647-62.
